# Scalable nanopatterning of organic light-emitting diodes beyond the diffraction limit

**DOI:** 10.1038/s41566-025-01785-z

**Published:** 2025-10-31

**Authors:** Tommaso Marcato, Jiwoo Oh, Zhan-Hong Lin, Tian Tian, Abhijit Gogoi, Sunil B. Shivarudraiah, Sudhir Kumar, Ananth Govind Rajan, Shuangshuang Zeng, Chih-Jen Shih

**Affiliations:** 1 Institute for Chemical and Bioengineering, ETH Zürich, Zürich, Switzerland; 2 Department of Chemical and Materials Engineering, University of Alberta, Edmonton, Alberta Canada; 3 Department of Chemical Engineering, Indian Institute of Science, Bengaluru, India; 4 School of Integrated Circuits, Huazhong University of Science and Technology, Wuhan, China

**Keywords:** Organic LEDs, Nanophotonics and plasmonics

## Abstract

Miniaturization of light-emitting diodes below the diffraction limit of the emission wavelength can enable super-resolution imaging and on-chip light sources for ultrabroadband chiplet communication. Organic light-emitting diodes, although suitable for miniaturization due to their emission from localized excitons, suffer from the limited compatibility of organic materials with traditional photolithographic patterning. Here we develop a method for the scalable fabrication of nanoscale organic light-emitting diodes with pixel densities up to 100,000 pixels per inch, periodicity of 250 nm and the smallest pixel size in the order of 100 nm. We realize the direct nanoscale patterning of organic semiconductors by self-aligned nanostencil etching and lithography. The process is resist-free and involves etching and evaporation through nanoapertures in a free-standing film adhering to the substrate. A nanoscale organic light-emitting diode surface with over 1 megapixel exhibits an average external quantum efficiency of 13.1%. We also demonstrate electroluminescent metasurfaces with subwavelength-scale meta-atoms that can electrically modulate the emitted light. The diffractive coupling between nanopixels enables control over the far-field emission properties of light, including directionality and polarization. These results pave the way for hybrid integrated photonics technologies, including visible-light communication, lasing and high-resolution displays.

## Abstract (web summary)

Nanostencil etching and lithography enable the fabrication of green-emitting nanoscale organic light-emitting diode pixels with size as small as 100 nm, densities as high as 100,000 pixels per inch and average external quantum efficiency of 13.1% for green emission.

## Main

Miniaturization is the trend to manufacture ever-smaller devices and products. The fabrication of small light-emitting diodes (LEDs)—a category of semiconductor devices that convert electricity into light—is at the forefront of research and development^1–3^. Emerging technologies such as ultrabroadband on-chip communication^4–7^ demand submicrometre-scale LEDs operating in the visible and infrared wavelength regions^8–10^. Inorganic III–V LED technology has been considered a promising candidate. However, inorganic LEDs suffer from size-dependent external quantum efficiency (EQE) reduction, which substantially compromises electroluminescence (EL) performance due to the increase in non-radiative recombination defects introduced during the semiconductor patterning process^11–17^.

In this regard, an overlooked advantage of using organic emitting materials is the fact that their emission arises from Frenkel excitons, which are highly localized within individual molecules in amorphous films^18^. This intrinsic property makes organic-light emitting diode (OLED) technology fundamentally suitable for miniaturization^19^. Despite their widespread use in large-area displays^20–22^, the fabrication of small OLED pixels remains challenging, due to the incompatibility of organic materials with conventional microfabrication processes based on photolithography^18^. In commercial OLED manufacturing, the patterning of organic semiconductors relies on the direct evaporation of organic molecules through a fine metal mask. However, the large thickness of fine metal masks prevents the patterning of fine (<10 µm) features. Several studies have demonstrated submicrometre OLED pixels by confining the bottom-electrode area using insulating photoresists or electron-beam resists, without patterning the emissive layer (EML)^23–25^. However, these approaches often suffer from limited scalability and throughput and, most critically, do not permit the isolation of organic emitting patterns—an essential capability for advanced applications such as full-colour multiplexing.

**Fig. 1:**
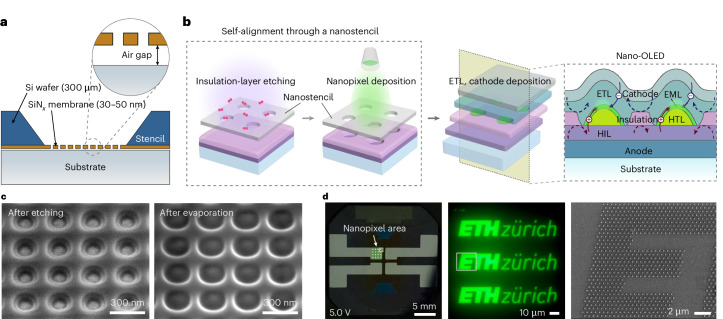
Scalable fabrication of nano-OLEDs. **a**, Cross-sectional schematic of a nanostencil in close contact with the substrate. The free-standing SiN_*x*_ membrane faces the substrate, separated by an air gap of 0.5–1 µm. **b**, Illustration of the nano-OLED fabrication process. After spin coating the HIL and the insulation layer, a nanostencil is aligned and attached to the substrate, serving as a mask for etching and thermal evaporation of the organic nanopixels, consisting of the HTL and EML, resulting in self-aligned pixel insulation. Subsequent layers, ETL and cathode, are deposited as continuous films. **c**, SEM images of the nanopixels at different steps of the fabrication process. The nanostencil features a square array of circular nanoapertures with a diameter of 100 nm and a periodicity of 300 nm. **d**, Photograph of a nano-OLED device operating at 5.0 V (left), and the optical micrograph (middle) of a nanopixel array displaying the ‘ETH Zürich’ logo at 50,000 ppi. Each logo comprises approximately 2,800 nanopixels, and the SEM image (right) shows a part of the nanopixel array. The nanodisc OLED pixels have a diameter of 200 nm and a periodicity of 500 nm.

**Extended Data Fig. 1:**
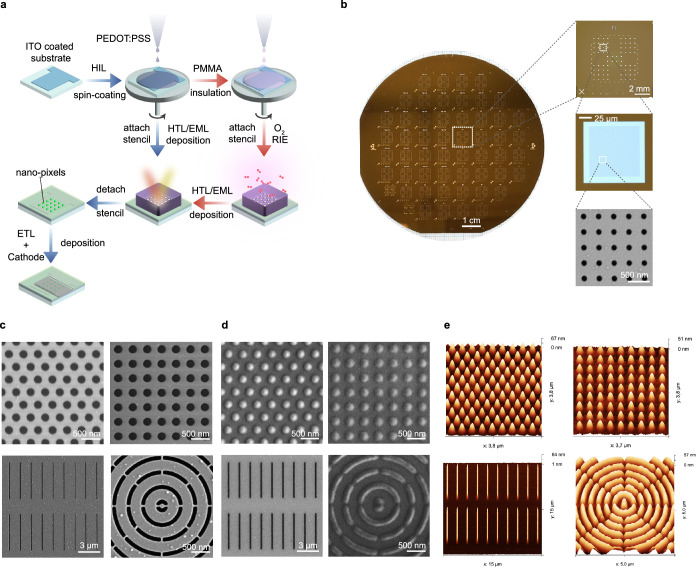
Nano-OLED Fabrication. **a**, Schematic diagram illustrating the fabrication procedure for the bottom-emitting Nano-OLEDs. The additional process following the red arrows yields the configuration “Insulation”. **b**, Photographs of a 4-inch nanostencil wafer containing 52 chips. Each chip has 64 freestanding windows made by nanoaperture SiN_x_ membrane of dimension of 100 × 100 µm^2^. Scale bars in right micrographs (top to bottom): 2 mm; 25 µm; 500 nm. **c-d**, Representative SEM images for the nanostencils (**c**) and resulting nanopatterns of organic semiconductors (**d**). **e**, Representative AFM images for the deposited nanopatterns of organic semiconductors.

To address the need for high-resolution, scalable patterning of organic semiconductors for miniaturized OLED applications, we present a process for the fabrication of nano-OLED pixels with pixel densities of up to 10^5^ pixels per inch (ppi), with individual pixel dimensions down to approximately 100 nm. This is achieved through the direct nanopatterning of solvent-sensitive organic EMLs by using self-aligned nanostencil etching and lithography. Nanostencil lithography^26–29^ is a resist-free, bottom-up patterning technique that involves shadow-mask evaporation through nanoapertures on a free-standing membrane, referred to as a nanostencil, and that has recently been proposed as a promising approach for OLED miniaturization^30,31^. The membrane is ultrathin (down to 30 nm) and corrugation-free over a large area (>100 µm), allowing the nanostencil to adhere closely to the substrate surface with a uniform and consistent air gap of 1–2 µm, thereby enabling the precise transfer of nanopatterns (Fig. 1a). The nanostencil carries the predefined nanopatterns that are transferred onto the substrate as active emitting areas or nanopixels (Extended Data Fig. 1c–e). The nanostencil chips can be attached and removed without damaging the substrate, and reused multiple times. Furthermore, our method eliminates the need for a complicated patterning process for each device, substantially improving throughput and reducing fabrication time and cost.

## Scalable fabrication of nano-OLEDs

Nanostencil fabrication begins with the deposition of an ultrathin (30–50 nm) silicon nitride (SiN_*x*_) film grown on a silicon (Si) wafer using low-pressure chemical vapour deposition. Nanoapertures in the SiN_*x*_ film are patterned by electron-beam lithography, followed by selective reactive ion etching (RIE). The SiN_*x*_ membrane is then released from the supporting Si substrate through a combination of dry and wet Si etching (Methods and Supplementary Fig. 1). Extended Data Fig. 1b shows a four-inch Si wafer containing 52 nanostencil chips, each of which includes 64 free-standing SiN_*x*_ membranes with different nanopatterns.

We optimized the nano-OLED device fabrication process by using the nanostencil as a mask for self-aligned etching and deposition. Figure 1a shows a nanostencil attached to a substrate, with the membrane making direct contact with the surface. Despite this firm contact, an air gap of around 1–2 μm is always present (Supplementary Figs. 10–12 and Supplementary Table 9). Figure 1b summarizes the key steps of the fabrication process. First, a nanostencil is aligned and attached to a stack consisting of an indium tin oxide (ITO) anode, poly(3,4-ethylenedioxythiophene)-poly(styrenesulfonate) (PEDOT:PSS; hole injection layer (HIL)) and poly(methyl methacrylate) (PMMA; insulation layer). Once the nanostencil is aligned and attached to the substrate, RIE is performed using oxygen plasma. Etchant gas molecules that diffuse through the nanoapertures enable the selective removal of the underlying insulation layer, thereby exposing the HIL. Subsequently, di-[4-(N,N-di-*p*-tolyl-amino)-phenyl]cyclohexane (TAPC; hole transport layer (HTL)) and tris(2-phenylpyridine)iridium(III) (Ir(ppy)3):4,4’-bis(N-carbazolyl)-1,1’-biphenyl (CBP; EML) are deposited through the nanostencil. Finally, 4,6-bis(3,5-di-3-pyridinylphenyl)-2-methylpyrimidine (B3PymPm; electron transport layer (ETL)), 8-hydroxyquinolinolato-lithium (Liq; electron injection layer (EIL)) and aluminium (Al; cathode) are deposited as continuous films after the nanostencil is detached from the substrate. The self-aligned process presented here demonstrates the first scalable approach to achieve both single-nanopixel patterning and electrical insulation. In addition, it is directly compatible with conventional OLED fabrication processes, requiring no additional nanofabrication or alignment steps. The pixel insulation effectively blocks the leakage current that would otherwise arise from direct contact between the HTL and ETL outside the device emission area (Fig. 1b shows the schematic cross-section), thereby maximizing the device performance.

Figure 1c presents the scanning electron microscopy (SEM) images of the nano-OLED device at different steps of the fabrication process, after performing self-aligned RIE (left) and thermal evaporation of EML (right). Figure 1d shows a representative working device. The nanostencil is aligned such that each addressable unit contains 16 nanopixel areas (Supplementary Fig. 23 shows a schematic of the layout). Because the nanoapertures are defined by electron-beam lithography, the array design and geometry are highly customizable. In this demonstration, the individual nanopixels are nanodisc OLED devices arranged in a hexagonal array that displays the ETH Zürich logo, with a nanodisc diameter of 200 nm and periodicity of 500 nm.

## Nanomolecular patterning of organic semiconductors

**Fig. 2:**
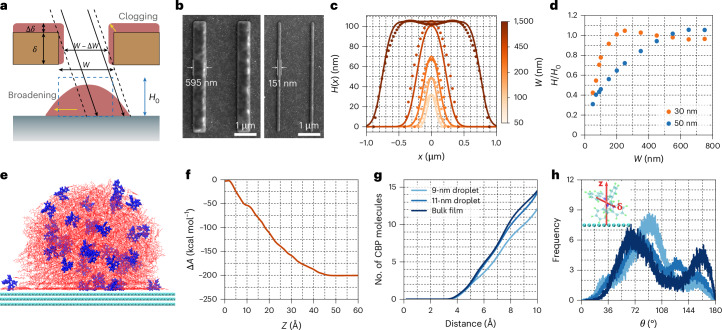
Nanomolecular patterning of organic semiconductors. **a**, Schematic of non-ideal effects in the deposition of nanomolecular patterns through a nanoaperture. **b**, Representative SEM images of the deposited organic nanorods with widths of 151 and 595 nm, by thermal evaporation through slit nanoapertures with widths of 50 and 550 nm, respectively, onto a PEDOT:PSS-coated glass substrate. **c**, AFM-characterized height profiles (dots) of nanorods as a function of lateral position for various slit widths, compared with our simulations (solid lines). **d**, AFM-characterized height of nanorod structures normalized by the target height, *H*/*H*_0_, as a function of *W*. The two datasets correspond to two different nanostencil thicknesses—30 and 50 nm. **e**, Molecular-dynamics-simulated post-equilibrium snapshot of a nanodroplet comprising Ir(ppy)_3_:CBP on a graphite surface, showing the accumulation of Ir(ppy)_3_ molecules (blue) at the surface. The diameter of the nanodroplet is approximately 11 nm. **f**, Calculated Helmholtz free energy change (Δ*A*) as a function of migration distance (*Z*), corresponding to the movement of a single Ir(ppy)_3_ molecule from the centre of a free-standing Ir(ppy)_3_:CBP droplet (*Z* = 0) to the droplet–vacuum interface (*Z* ≈ 4.5 nm). **g**, Average number of CBP molecules coordinating each Ir atom in Ir(ppy)_3_ molecules within nanodroplets of two different diameters (9 and 11 nm) and of the bulk Ir(ppy)_3_:CBP film. **h**, Angular distribution of Ir(ppy)_3_ TDM (**δ**) relative to the substrate normal vector (**z**).

One of the most important breakthroughs for the fabrication protocol presented here is the demonstration of the nanomolecular patterning of organic semiconductors. We examined this capability by depositing a 100-nm-thick layer of the organic semiconductor TAPC through a nanostencil chip containing slit-shaped nanopertures with widths (*W*) ranging from 50 to 1,500 nm. As revealed in Fig. 2a, although deposition from a uniform perpendicular molecular flux is expected to produce patterns with lateral dimensions identical to the aperture size (blue dashed rectangle), there are two non-ideal effects influencing the actual pattern formation (yellow arrows).

Specifically, first, the width of the deposited nanorod pattern tends to be broader than that of the aperture owing to the divergence of the molecular beam generated from an evaporation source^32^. The pattern broadening effect is governed by geometrical factors and becomes more pronounced with increased nanostencil–substrate separation and nanostencil–source distance. Second, due to the finite thickness of the nanostencil, part of the angle-dependent molecular flux is obstructed by the sidewalls of the nanoapertures (self-shadowing). This leads to undesirable deposition on the sidewalls, reducing the effective slit width (*W* – Δ*W*) and increasing the effective membrane thickness (*δ* + Δ*δ*; Fig. 2a). This clogging effect becomes particularly considerable when the nanoaperture dimension is comparable with the nanostencil thickness: *W* ≈ *δ*. Figure 2b presents the representative SEM images of the nanorods deposited through slit nanoapertures with nominal widths of 500 nm (left) and 50 nm (right), which result in actual nanorod widths of 595 and 151 nm, respectively.

Accounting for these non-ideal effects, we have developed a theoretical model^33^ for the prediction of height profiles of organic semiconductors deposited through nanostencils, considering (1) the angular distribution of molecular flux, (2) geometrical factors and (3) the clogging effect (Supplementary Discussion 1). Figure 2c compares the atomic force microscopy (AFM) profiles with the simulated profiles for various slit widths, *W*. For narrow slits (*W* ≈ *δ*), a combination of clogging and self-shadowing results in nanorods that are shorter than the target height ${H}_{0}$. In the case of the narrowest slit (50 nm), the resulting nanorods exhibit a parabolic cross-sectional profile and reach only about 30% of the target height (Supplementary Fig. 13). As *W* increases (*W* $\gg$ *δ*), the cross-sectional profiles of the nanorods change to a more trapezoidal shape, with the flat central region converging to the target height ${H}_{0}$.

Figure 2d presents the AFM-characterized maximum height of the nanostructures as a function of *W* for two different nanostencil thicknesses. When using a thinner stencil (*δ* = 30 nm), the clogging effect is notably reduced, particularly for narrower slits, allowing the nanorods thickness to reach the target height at *W* ≥ 200 nm. By contrast, the target height is reached only for *W* ≥ 500 nm with the thicker nanostencil (*δ* = 50 nm).

To investigate how nanomolecular patterning influences the molecular behaviour within nano-confined EL (Ir(ppy)_3_:CBP) with a high surface-to-volume ratio, we performed large-scale all-atom molecular dynamics simulations of nanodroplets composed of an Ir(ppy)_3_:CBP molecular blend (Supplementary Discussion 3). During the simulations, we observed that the Ir(ppy)_3_ molecules preferentially migrate towards the periphery of supported nanodroplets (Fig. 2e). To quantify this behaviour, we computed the Helmholtz free energy change (Δ*A*) associated with relocating a single Ir(ppy)_3_ molecule from the centre of a free-standing droplet to the droplet–vacuum interface. The results show a monotonic decrease in Δ*A* (Fig. 2f), indicating that this migration is thermodynamically favourable. These findings suggest that Ir(ppy)_3_ molecules exhibit surface activity within the CBP matrix.

The surface activity of Ir(ppy)_3_ results in two notable effects. First, we compared the average number of CBP molecules coordinating each Ir atom within the nanodroplets of two different diameters (9 and 11 nm) and the bulk film (Fig. 2g). Relative to the bulk film, the number of CBP molecules surrounding individual Ir(ppy)_3_ molecules decreases as the droplet size becomes smaller. Second, we analysed the angular distribution of the Ir(ppy)_3_ transition dipole moment (TDM) direction **δ**, relative to the substrate normal vector **z** (Fig. 2h). The mean angles were found to be 88.81°, 94.88° and 90.79°, for the bulk film, 11-nm-diameter droplet and 9-nm-diameter droplet, respectively. The calculated distributions allow us to estimate the ensemble average of the square cosine of the TDM angles, yielding values of 0.37, 0.30 and 0.24 for the bulk film, 11-nm droplet and 9-nm droplet, respectively. These values can be compared with that expected for an isotropic orientation (0.33), suggesting that nanomolecular confinement promotes a certain degree of vertical TDM alignment. Overall, our findings imply that nanopatterning the EML can alter the ensemble TDM orientation. Future investigations integrating experimental measurements and molecular modelling are required to fully understand the effects of nanoconfinement.

## Electroluminescent characteristics of nano-OLEDs

**Fig. 3:**
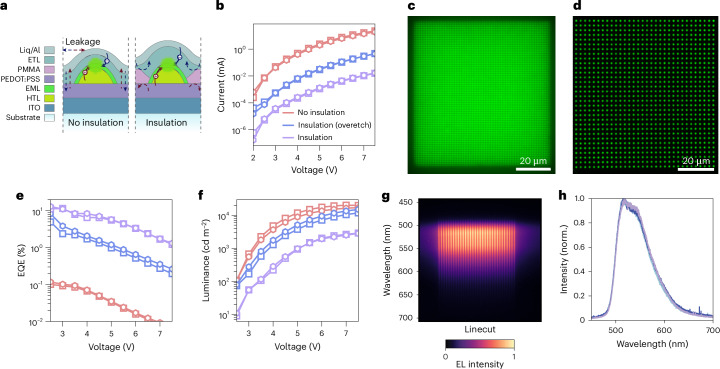
Nano-OLED EL characteristics. **a**, Schematics comparing the insulation and no-insulation device configurations for the optimization of nano-OLED performance. **b**,**e**,**f**, Effects of pixel insulation on nano-OLED performance, comparing the current (**b**), EQE (**e**) and luminance (**f**) as a function of voltage. The devices considered here are square (square symbols) and hexagonal (hexagonal symbols) arrays of nanodisc OLED pixels of 100-nm diameter and 350-nm pitch. **c**,**d**, Representative optical micrographs of operating nano-OLEDs at 25,000 ppi (**c**) and 10,000 ppi (**d**), showing uniform emission intensity over the whole nanopixel area. **g**, Extraction of EL spectra for individual pixels along one column of the square array of nanodisc pixels in **d**. **h**, Comparison of normalized (norm.) EL spectra of four representative pixels, which are all consistent with those for bulk thin-film devices.

**Extended Data Fig. 2:**
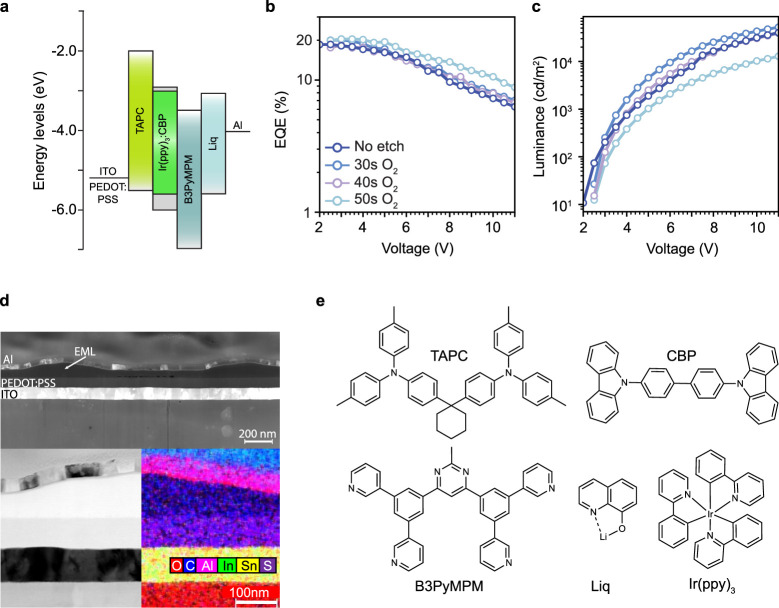
OLED Reference Device and Architecture. **a**, Schematic diagram illustrating the energy levels of organic layers in the bulk OLED devices. The EQE (**b)** and luminance (**c**) responses as a function of voltage for the Ir(ppy)_3_:CBP reference thin film OLED devices (No etch), in comparison with those having the PEDOT::PSS layers exposed to varied O_2_ RIE durations (0, 30, 40 and 50 seconds). Performance data are summarized in Table S10. **d**, Cross-sectional TEM images and EDS map of a representative Nano-OLED device consisting of a 1D linear array of 300 nm wide nanorods with 1 µm periodicity. **e**, Chemical structures of the materials used in our optimized OLED architecture.

The optimized protocol for nanomolecular patterning was directly incorporated into the fabrication of bottom-emitting nano-OLEDs on glass substrates, based on our benchmark device architecture of ITO/PEDOT:PSS/TAPC/Ir(ppy)_3_:CBP/B3PymPm/Liq/Al (Fig. 3a shows the ‘no-insulation’ configuration; Extended Data Fig. 2 shows the device information and cross-sectional transmission electron microscope (TEM) image and energy-dispersive X-ray spectroscopy (EDS) map).

As introduced earlier, this basic configuration suffers from leakage currents due to the lack of electrical insulation between pixels, limiting the EQE to 2%–3%. To overcome this, we applied our self-aligned nanostencil etching strategy to selectively remove the insulation layer at the emission sites, enabling single-pixel insulation and substantially suppressing leakage currents (Fig. 1b; see the ‘insulation’ configuration in Fig. 3a).

The scalable fabrication of nano-OLEDs enables the precise characterization of their performance using the same instrumentation as for large-area planar LEDs. Figure 1d presents a representative photograph of an operating nano-OLED device used for EQE measurements, with simultaneous emission from 1.45 megapixels. Figure 3c,d shows the EL micrographs of nano-OLED designs with larger periodicities, corresponding to 25,000 and 10,000 ppi, respectively, to allow a clear resolution of individual pixels. Additional micrographs of photoluminescence (PL) and EL from nano-OLEDs are provided in Supplementary Figs. 19–21. It is important to note that PL in nano-OLEDs originates solely from the nanopatterned EML, whereas EL also involves charge transport and injection at HTL–EML interface. Variations in interfacial stacking and layer uniformity strongly affect the spatial uniformity of EL emission, making EL inherently less uniform than their PL across the device area.

**Fig. 4:**
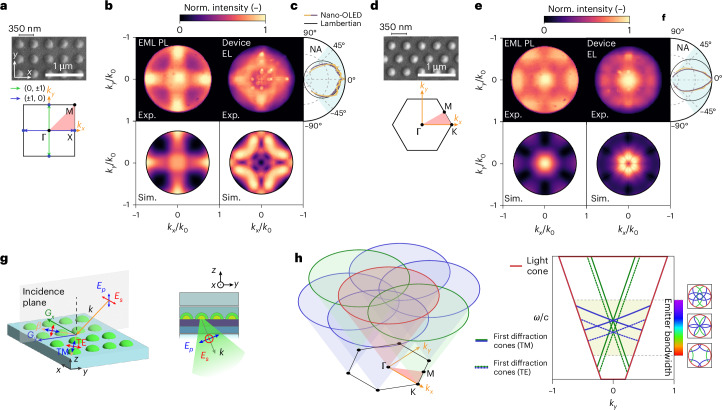
Controlling emission directionality of electroluminescent metasurfaces made by 2D arrays of nanodisc OLED pixels. **a**,**d**, SEM images and first Brillouin zones for 2D square (**a**) and hexagonal (**d**) arrays of EML nanodiscs deposited on glass. **b**,**e**, Experimental (Exp.) and simulated (Sim.) BFP images for PL of nanopatterned EML (left) and device EL (right) arrays with square (**b**) and hexagonal (**e**) lattice. The EL BFP images of the electroluminescent metasurfaces (right) reveal that the square array directs light to high angles ($0.3 < {{|k}}_{\parallel }| < 0.8$) and the hexagonal array concentrates light at low angles (${{|k}}_{\parallel }| < 0.25$). **c**,**f**, Polar plots for the corresponding angular dependence of EL intensity extracted from the vertical (purple) and horizontal (yellow) cuts in the BFP images of square (**c**) and hexagonal (**f**) arrays. The cuts are corrected by the $\cos \theta$ apodization factor and compared with the Lambertian profiles (black) expected from planar OLEDs of uniform EML. **g**, Schematics showing the experimental frames of reference with respect to the array axis and the directions of polarizer slit for optical characterization. *E*_*p*_ and *E*_*s*_ are the projections of the electric field in the plane of incidence and in the plane perpendicular to it, respectively. **h**, Schematic illustrating how the reciprocal lattice of the electroluminescent metasurfaces determines the radiation pattern. The response of the array in *k* space is equal to the convolution of the free photon dispersion (the light cone) and the reciprocal lattice (the intersection of light cones centred at the reciprocal lattice points). Each frequency component of the emitter spectrum contributes to a horizontal isofrequency slice of the array response, resulting in a broadening of the sharp circular modes. The right energy–momentum dispersion illustrates the first-order diffraction. Depending on the angle between the grating vector **G** and the *k*_*x*_ axis, the vertical *ω*–*k*_*x*_ plane cuts the cones in the lattice dispersion resulting in either linear or hyperbolic photonic bands.

Device characteristics were, therefore, evaluated and compared across three basic configurations (Fig. 3b,e,f). The optimal array design was selected based on their radiation pattern (see the ‘Electroluminescent metasurfaces with controlled emission directionality’ section and Fig. 4). Alongside no insulation, the second configuration (blue) includes a PMMA insulation layer that was overetched, suppressing leakage between neighbouring arrays without providing sidewall separation between individual pixels. The third and ideal configuration fully uses the self-aligned pixel insulation illustrated in Fig. 1b, with the non-emissive area fully covered by the PMMA layer. As shown in Fig. 3b, current decreases with increasing degree of insulation, due to reduced current leakage in the non-emissive areas. At the device turn-on voltage (2.3 V), the current is reduced by three orders of magnitude compared with the no-insulation case.

However, the large-area overlay between the insulating barrier and organic nanopatterns demands accurate alignment between oxygen plasma and molecular fluxes through the membrane nanoapertures. With optimized RIE and HIL deposition protocols, we achieved maximum EQE values of 11.6% and 13.1% for square and hexagonal arrays, respectively (Fig. 3e), in the self-aligned insulation configuration. Despite the high EQE values, the maximum luminance (reaching up to ~4,000 cd m^−2^) remains modest, indicating room for further optimization of the single-pixel insulation step.

## Electroluminescent metasurfaces with controlled emission directionality

The nanoscale patterning of organic semiconductors enables unprecedentedly small pixel dimensions (<*λ*/4) and periodicities (<*λ*/2) at visible frequencies. Access to subwavelength scales shifts the operating regime of OLED device into wave optics, where individual nanopixels act as meta-atoms forming electroluminescent metasurfaces^34^. For a proof-of-concept demonstration, we focus on the control of two crucial aspects of OLED emission relevant to technological applications: EL directionality and polarization. To this end, we designed three families of electroluminescent metasurfaces: (1) two-dimensional (2D) periodic arrays of nanodiscs for directional emission, (2) one-dimensional (1D) circular gratings (bull’s-eyes) for omnidirectional radiation pattern engineering and (3) 1D linear arrays of nanorods for the control of the degree of linear EL polarization. For each pattern, we examined the far-field back focal plane (BFP) PL/EL images of pixelized EML deposited on bare glass substrates and bottom-emitting nano-OLED devices fabricated on glass, using a high-numerical-aperture (NA) Fourier microscope^35,36^.

Radiation generated by the nano-OLED device propagates through space as spherical waves with a distinct angular distribution of intensity, polarization and energy. Each angle up to the objective’s NA, corresponding to an in-plane photon momentum $\left({k}_{x},{k}_{y}\right)={k}_{0}\left(\sin \theta \cos \phi ,\sin \theta \sin \phi \right)$, where ${k}_{0}=2{\rm{\pi }}/\lambda$ is the free-space momentum of photon with wavelength *λ*, is collected and mapped onto the BFP of the microscope objective, generating an image effectively equivalent to the spatial Fourier transform of the front focal plane image (Methods).

For the first family of electroluminescent metasurfaces, we designed the lattice periodicity $p=350$ nm to match the Ir(ppy)_3_ emission maximum to the second Bragg condition of the lowest waveguided mode of the OLED stack, where the local density of optical states at the Γ point, $\left|{k}_{\parallel }\right|=0$ (normal incidence), is maximized. To decouple the BFP images from contributions of the OLED stack, we first analysed PL from metasurfaces comprising nanodisc arrays of EML deposited on glass substrates (reference frame, Fig. 4g (left)). The nanodisc arrays can be modelled as square and hexagonal photonic crystal lattices having $x-y$ periodic modulation of the refractive index (SEM images and Brillouin zones are shown in Fig. 4a,d), which determines the electromagnetic Bloch modes in the associated photonic band structures. The nanodiscs radiate incoherently across a broad range of wavevectors and couple to Bloch modes under phase-matching conditions: the in-plane wavevector ${k}_{\parallel }$ is conserved modulo *G*, that is, $\left|{k}_{\parallel }\right|=\left|{k}_{\parallel }^{{\rm{in}}}+{\bf{G}}\right|$, where ${\bf{G}}=2{\rm{\pi }}/p$ is the reciprocal lattice vector or the ‘grating’ momentum, and ${k}_{\parallel }$ and ${k}_{\parallel }^{{\rm{in}}}$ are the wavevectors of the output and input modes, respectively. Consequently, the reciprocal lattice of the pixel array modulates the far-field radiation pattern within the light cone, that is, $\left|{k}_{\parallel }\right| < {k}_{0}$ (Fig. 4h)^37^. This effect is clearly visible in their PL BFP images (EML PL images are shown in Fig. 4b,e), whose rotational symmetries directly reflect the point groups of the corresponding reciprocal lattices.

**Extended Data Fig. 3:**
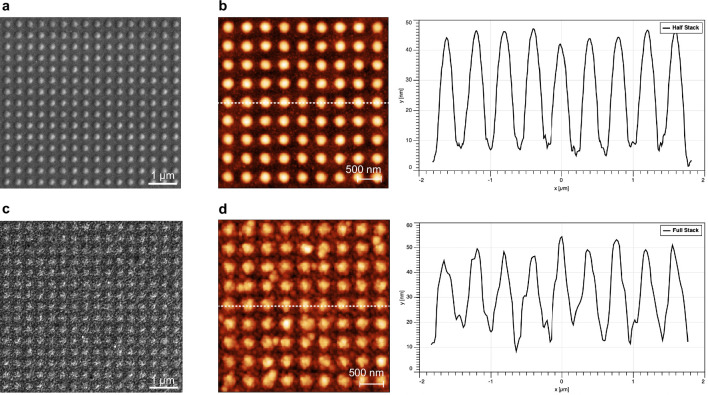
Representative SEM and AFM images of Nano-OLED. half-stack (**a** and **b**) and full-stack (**c** and **d**) structures illustrating the transfer of the EML pattern to the top Al cathode layer. Height profiles corresponding to the dashed linecuts are shown on the right.

**Extended Data Fig. 4:**
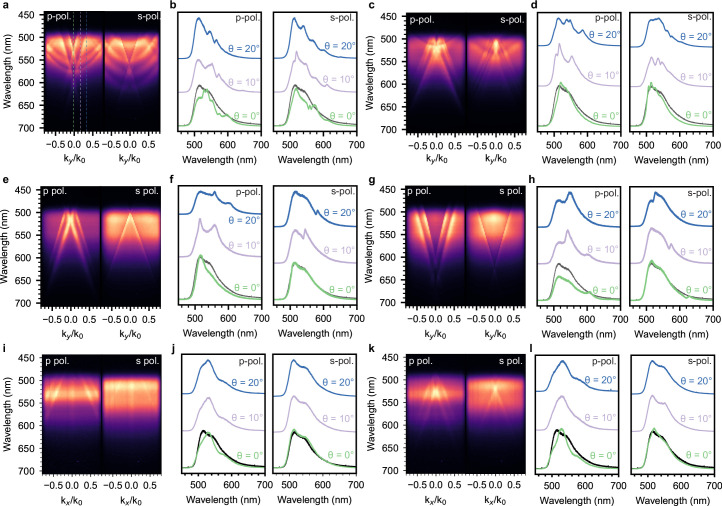
Angle-dependent Spectra of electroluminescent metasurfaces considered in this study. **a-n** Angle-resolved PL spectra for the devices in Figs. 4 to 6, including 2D square (**a**), hexagonal (**c**) and 1D linear (**e** and **g**) pixel arrays along the Γ-Χ, Γ-Κ directions in the Brillouin zone, and k_y_ direction, respectively, as well as the bullseye EML patterns **(i** and **k)** with the polarizer parallel (*p*-pol.) and perpendicular (*s*-pol) to the spectrometer slit. Spectra corresponding to line cuts at 0^o^, 10^o^, and 20^o^ (**b, d**, **f, h, j**, and **l**) were extracted to resolve the TM (*p*-polarized; *p*-pol) and TE (*s*-polarized; *s*-pol) modes. The gray curves correspond to PL spectra for the uniform EML film.

The EL BFP images of the full nano-OLED devices (the device EL images are shown in Fig. 4b,e) involve additional complexity due to the existence of plasmonic and waveguided modes (reference frame, Fig. 4g (right)). These modes, which are otherwise inaccessible, can be diffracted by the grating into free-space photons as the geometry of the EML nanostructures is transferred to the topmost layers (Extended Data Fig. 3). The angle-dependent PL spectra shown in Extended Data Fig. 4 and simulations shown in Supplementary Fig. 26 allow us to distinguish these modes by their characteristic dispersions. The device architecture supports two transverse-magnetic (TM), or *p*-polarized, modes: (1) a broader resonance at lower frequency, consistent with a plasmonic mode of the Al cathode, and (2) a narrow TM_0_ waveguided mode of the ITO anode. According to the electric field profiles (Supplementary Fig. 5), the latter mode hybridizes into a waveguide-plasmon polariton^38,39^. Additionally, the *s*-polarized mode corresponds to the transverse-electric (TE_0_) waveguided mode of the ITO anode. In both PL and EL, the emission from the nano-OLED metasurfaces arises from the coupling between the broad spectrum of the organic emitter and narrowband grating modes, resulting in asymmetric spectral features consistent with Fano-type interference^40^ (Extended Data Fig. 4g).

Remarkably, despite the broadband emission spectrum of Ir(ppy)_3_, the simulated and experimental BFP images of the device stack (Fig. 4e,c) reveal that the majority of radiation is concentrated within $\left|{k}_{\parallel }\right| < 0.25{k}_{0}$ or ±15° in air. By contrast, the electroluminescent metasurface with square arrays (Fig. 4b,f) directs light to higher angles, $0.3 < {{|k}}_{\parallel }| < 0.8$, as the outcoupled modes move towards the Χ point.

**Fig. 5:**
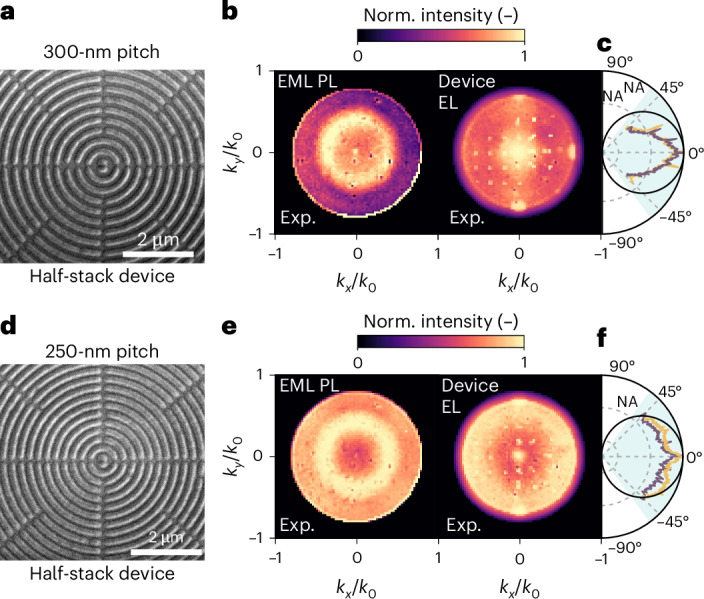
Controlling omnidirectional EL of electroluminescent metasurfaces made by concentric bull’s-eye nano-OLED pixels. **a**,**d**, SEM images of 1D concentric bull’s-eye nanopatterned EML with *p* values of 300 nm (**a**) and 250 nm (**d**). **b**,**e**, BFP images for the PL of nanopatterned EML (left) and device EL (right) of the bull’s-eye patterns in **a** (**b**) and **d** (**e**), showing radiation patterns independent of the azimuthal angle. The EL radiation patterns reveal that tuning the bull’s-eye periodicity redirects emission to different polar angles *θ*. **c**,**f**, Polar plots for the angular dependence of EL intensity extracted from the vertical (purple) and horizontal (yellow) cuts in the BFP images of the patterns in **a** (**c**) and **d** (**f**). The cuts are corrected by the cos*θ* apodization factor and compared with the Lambertian profiles (black).

**Extended Data Fig. 5:**
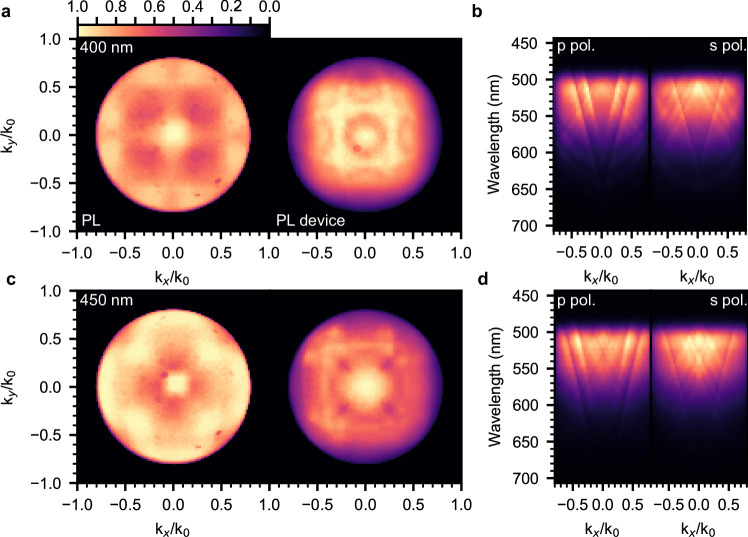
PL characteristics of the electroluminescent metasurfaces based on square arrays of nanodisk OLED pixels. **a,c** Experimental PL BFP images for bare nanopatterned EMLs deposited on glass and their corresponding electroluminescent metasurfaces with 400 nm (**a**) and 450 nm (**c**) periodicities. **b,d** Corresponding angle-dependent PL spectra along the Γ-Χ direction, with the polarizer parallel (*p*-pol.) and perpendicular (*s*-pol) to the spectrometer slit.

**Extended Data Fig. 6:**
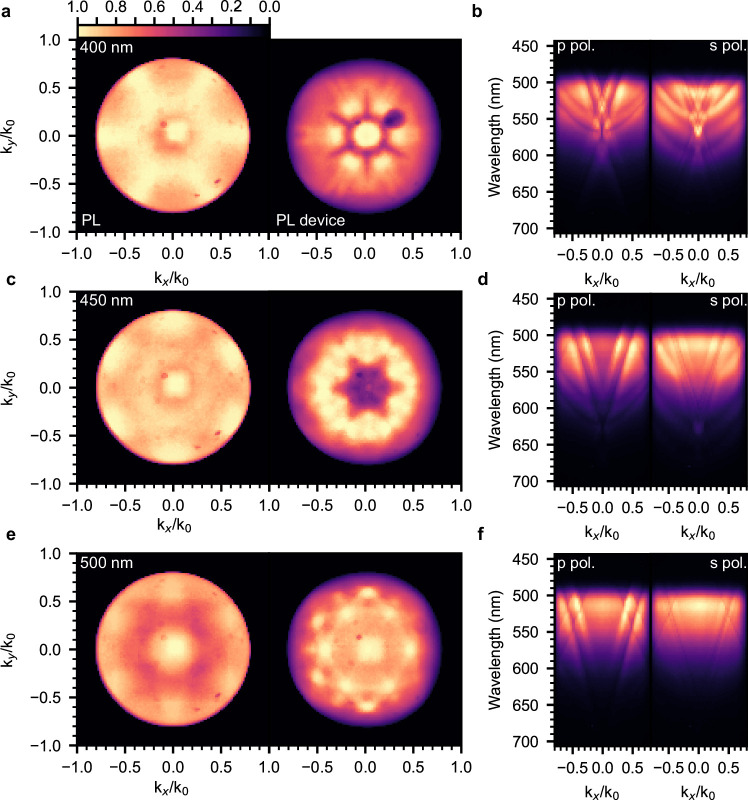
PL characteristics of electroluminescent metasurfaces based on hexagonal arrays of nanodisk OLED pixels. **a,c,e** Experimental PL BFP images for bare nanopatterned EMLs deposited on glass and their corresponding electroluminescent metasurfaces with 400 nm (**a**), 450 nm (**c**), and 500 nm (**e**) periodicities. **b,d,f** Corresponding angle-dependent PL spectra along the Γ-Χ direction, with the polarizer parallel (*p*-pol.) and perpendicular (*s*-pol) to the spectrometer slit.

However, in 2D periodic gratings, the symmetry of the radiation pattern is constrained by the discrete rotational symmetry of the reciprocal lattice. As a result, the emission-intensity maxima appear only at specific high-symmetry points in *k* space, producing discontinuous bands along the azimuthal direction. As shown in Fig. 5, to overcome this limitation, we fabricated electroluminescent metasurfaces based on the bull’s-eye patterns of EML with radial periodicity *p* (the SEM images are shown in Fig. 5a,d). Indeed, a bull’s-eye structure is essentially a circular grating that is azimuthally invariant, offering linear periodicity along the radial direction and enabling omnidirectional diffraction^41,42^. Figure 5b,e shows uniform EL radiation patterns in devices based on the bull’s-eyes of *p* = 300 nm and *p* = 250 nm, demonstrating omnidirectional EL emission. In particular, the smallest periodicity considered here, *p* = 250 nm, corresponds to a pixel array density of 10^5^ ppi, which is at least one order of magnitude larger than the state of the art in the literature. Additional arrays were fabricated and their BFP images and dispersions are reported in Extended Data Figs. 5 and 6.

## Electroluminescent metasurfaces with controlled emission polarization

**Fig. 6:**
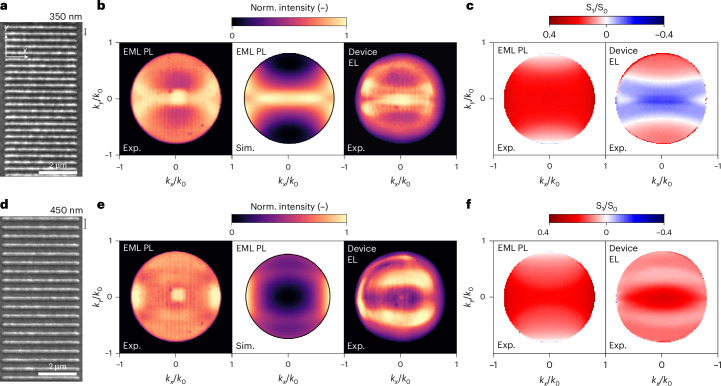
Controlling emission polarization of electroluminescent metasurfaces made by 1D arrays of nanorod OLED pixels. **a**,**d**, SEM images for 1D arrays of EML nanorods deposited on glass with *p* values of 350 nm (**a**) and 450 nm (**d**). **b**,**e**, Experimental (Exp.) and simulated (Sim.) radiation patterns of the structures in **a** (**b**) and **d** (**e**) that display emission directionality along the ${k}_{y}=0$ axis. **c**,**f**, Experimental maps of the ${{\rm{S}}}_{1}/{{\rm{S}}}_{0}$ Stokes parameter characterizing the degree of linear polarization as a function of in-plane momentum of the patterns in **a** (**c**) and **d** (**f**). The left maps exhibit linearly polarized PL along the nanorod long axis at all momenta for EML nanostructures on glass. On the contrary, the EL maps for the nano-OLED devices (right) reveal that the EL polarization is strongly influenced by leaky waveguide and plasmonic modes, such that the $p=350$ nm nanorod arrays yield *p*-polarized light (${{\rm{S}}}_{1}/{{\rm{S}}}_{0} < 0$) at low ${k}_{y}$, whereas the $p=450$ nm nanorod arrays remain to yield *s*-polarized light (${{\rm{S}}}_{1}/{{\rm{S}}}_{0} > 0$) at all angles. A change in the nanorod periodicity reverses the electroluminescent polarization at the perpendicular angle, $k=0$.

The third family of electroluminescent metasurfaces considered here are based on nanorod meta-atoms, which exhibit preferential emission polarization along their long axis at all angles (Fig. 6; details are shown in Supplementary Discussion 2 and Supplementary Fig. 4). For a complete characterization, we implemented angle-dependent polarimetry^43,44^ by adding a linear polarizer and a quarter-wave plate before the Fourier lens; therefore, the four Stokes parameters, namely, $${{\rm{S}}}_{0},$$ $${{\rm{S}}}_{1}$$, $${{\rm{S}}}_{2}$$ and $${{\rm{S}}}_{3}$$, at each $$({k}_{x},{k}_{y})$$ can be extracted by providing a full description of the polarization state of light in momentum space (Methods). The *s* and *p* polarizations refer to directions parallel and perpendicular to the nanorod’s axial axis, respectively. In particular, the parameter $${{\rm{S}}}_{0}$$ measures the total field intensity, whereas $${{\rm{S}}}_{1}$$ quantifies the degree of linear polarization with respect to the parallel ($${k}_{x}$$) and perpendicular ($${k}_{y}$$) bases, and it is calculated as $${{\rm{S}}}_{1}({k}_{x},{k}_{y})={I}^{s}\left({k}_{x},{k}_{y}\right)$$$$-{I}^{p}\left({k}_{x},{k}_{y}\right)$$, where $${I}^{s}$$ and $${I}^{p}$$ are the emission intensities from the *s*- and *p*-polarized BFP images, respectively. The ratio $${{\rm{S}}}_{1}/{{\rm{S}}}_{0}$$ quantifies the degree of linear polarization, ranging from +1 to −1: a more positive (negative) $${{\rm{S}}}_{1}/{{\rm{S}}}_{0}$$ value indicates preferential linear polarization along the parallel (perpendicular) basis, whereas unpolarized light yields $${{\rm{S}}}_{1}/{{\rm{S}}}_{0}=0$$ (refs. ^43,44^).

Analogous to the 2D nanodisc arrays discussed earlier, even though the emission generated from the nanopatterned EML is completely incoherent, the BFP images for the 1D arrays reveal that light is directed along the ${k}_{y}=0$ axis, orthogonal to the periodic direction via coherent scattering (Fig. 6b,e). The corresponding ${{\rm{S}}}_{1}/{{\rm{S}}}_{0}$ PL maps (Fig. 6c,f) exhibit preferentially parallel polarization at all angles. A lack of dependence on the array periodicity in the ${{\rm{S}}}_{1}/{{\rm{S}}}_{0}$ maps confirms that the observed linear polarization is dominated by response of the single nanorod.

**Extended Data Fig. 7:**
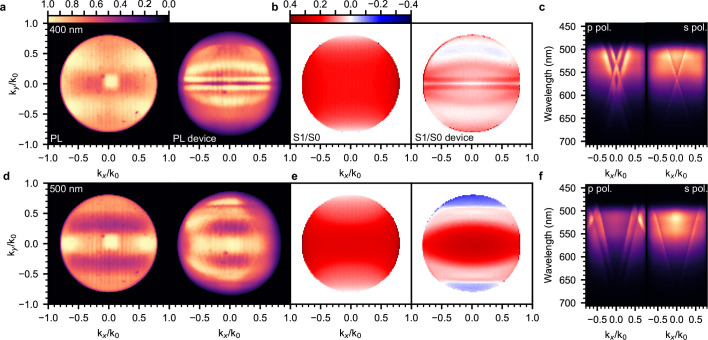
Characterization of PL polarization of electroluminescent metasurfaces based on linear arrays of OLED nanorod pixels. **a,d** Experimental PL BFP images of 1D linear arrays of 200 nm-wide nanorod EMLs deposited on glass and their corresponding electroluminescent metasurfaces with 400 nm (**a**) and 500 nm (**d**) periodicities. **b,e** Corresponding ${{\rm{S}}}_{1}/{{\rm{S}}}_{0}$ linear polarization maps. **c,f**, Corresponding angle-dependent PL spectra along the Γ-Χ direction, with the polarizer parallel (*p*-pol.) and perpendicular (*s*-pol) to the spectrometer slit.

**Extended Data Fig. 8:**
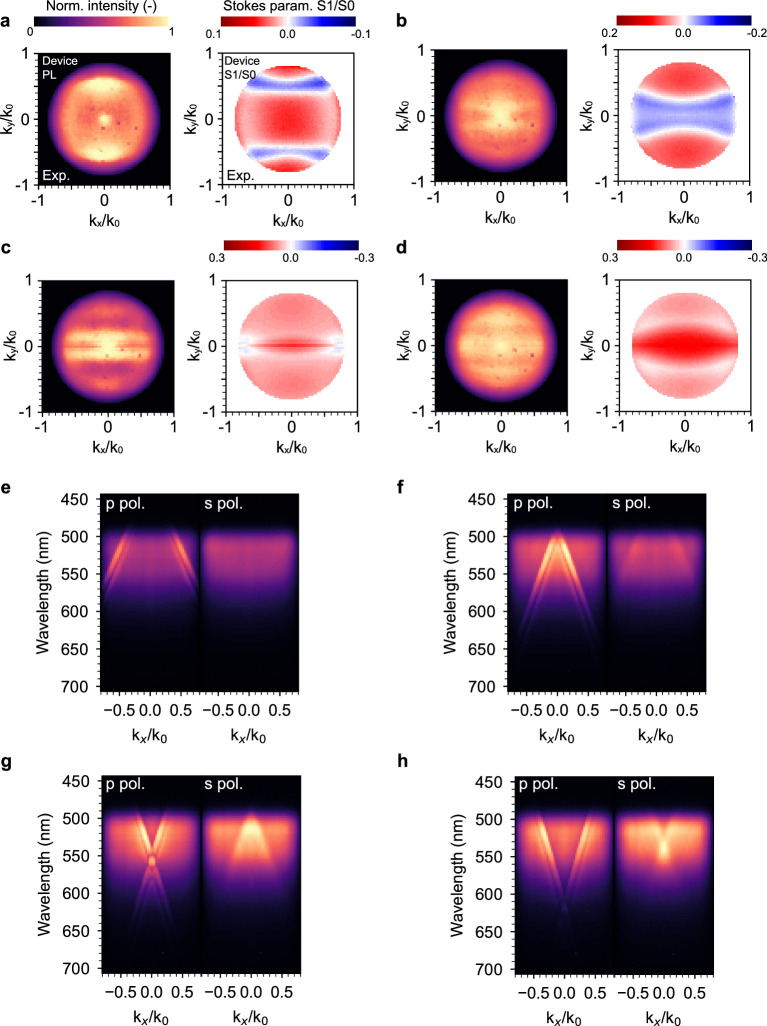
Characterization of PL polarization of electroluminescent metasurfaces based on linear arrays of OLED nanorod pixels. **a-d** Experimental PL BFP images and ${{\rm{S}}}_{1}/{{\rm{S}}}_{0}$ polarization maps of electroluminescent metasurfaces based on 1D arrays of 100 nm-wide nanorod pixels. The nanorod arrays have periodicities of 250 nm (**a**), 300 nm (**b**), 350 nm (**c**) and 400 nm (**d**). **e-h**, Corresponding angle-dependent PL spectra along the ${k}_{y}$ direction, with the polarizer parallel (*p*-pol.) and perpendicular (*s*-pol) to the spectrometer slit.

In nano-OLED devices, the polarization of leaky waveguide (TM_0_ and TE_0_) and plasmonic TM modes becomes relevant. As a result, the EL polarization response of 1D nano-OLEDs strongly depends on array periodicity. In particular, the electroluminescent metasurface formed by nanorod arrays of *p* = 350 nm (Fig. 6a) exhibits the opposite polarization (perpendicular, or *p* polarized, ${{\rm{S}}}_{1}/{{\rm{S}}}_{0} < 0$) for $\left|{k}_{y}\right| < 0.3$, due to the polarization of plasmonic and TM_0_ resonances. A high degree of perpendicular polarization (${{\rm{S}}}_{1}/{{\rm{S}}}_{0}$ = −0.25) is attained at zero angle, $k=0$, where EL is the strongest. On the other hand, the electroluminescent metasurface with *p* = 450 nm (Fig. 6d) displays a parallel polarization (*s* polarized, ${{\rm{S}}}_{1}/{{\rm{S}}}_{0} > 0$) at all momenta. Highly parallel-polarized light with ${{\rm{S}}}_{1}/{{\rm{S}}}_{0}$ of 0.4 is achieved at $k=0$. Remarkably, a variation in the periodicity of the 1D nanorod OLED array enables the reversal of far-field EL polarization (Extended Data Figs. 7 and 8).

## Conclusions

In summary, we have demonstrated the scalable fabrication of nano-OLEDs with pixel sizes and periodicities smaller than the diffraction limit of the emission wavelength, without compromising on device efficiency. Direct nanopatterning of emissive organic semiconductors gives rise to electroluminescent metasurfaces that convert injected charges into modulated light with controlled directionality and polarization. We anticipate that the ability to tailor the photonic landscape of organic semiconductors will open a new degree of freedom in OLED design, offering substantial advantages for emerging OLED technologies such as polariton OLEDs^45,46^, visible-light communication^47,48^ and lasing^49,50^.

## Methods

### Nanostencil fabrication

A double-side polished 300-µm-thick four-inch Si wafer was used as the substrate (Supplementary Fig. 1). A uniform layer of low-stress SiN_*x*_ (thickness, 30–50 nm) was deposited using low-pressure chemical vapour deposition (PEO-604, ATV Technologies).

Electron-beam lithography was then used to define nanopatterns on the SiN_*x*_ membrane (Supplementary Fig. 1b). An electron-beam resist (AR-P 6200.09, Allresist) was spin coated, followed by electron-beam exposure. Development was done using amyl acetate (AR 600-546, Allresist) as the developer and deionized water as the stopper. After development, the substrate is soft-baked for 2 min at 130 °C. Using the electron-beam resist as the mask, SiN_*x*_ is dry etched (Supplementary Fig. 1c) in an RIE chamber (PlasmaPro 80 RIE, Oxford Instruments). Anisole or AR 600-71 remover (Allresist) was used as a solvent for removing the electron-beam resist.

Photolithography was used to define the substrate back-side opening area (Supplementary Fig. 1d). After coating a photoresist (PR; AZ 10XT 520CP, MicroChemicals), PR exposure was performed by back-side alignment with a premade photomask using a mask aligner (EVG620 NT, EV Group). PR development was done using AZ 400K 1:4 (MicroChemicals) as a developer and deionized water as the stopper. Development was followed by a hard bake of 10 min at 115 °C. Before the back-side Si etching, the SiN_*x*_ membrane on the back side was etched using RIE (Supplementary Fig. 1e).

The Bosch process is used for deep Si etching in a Si deep RIE chamber (Omega Rapier, SPTS). PR was used as a mask. About 87% of the thickness of the Si wafer was etched using deep RIE. The remaining Si wafer (13% thickness) was etched using KOH wet etching. The wet etching was done in a water bath at 80 °C (Supplementary Fig. 1g).

### Device fabrication

Following materials were used for device fabrication: ITO, PEDOT:PSS, PMMA, TAPC, Ir(ppy)3, CBP, 4,6-bis(3,5-di(pyridin-3-yl)phenyl)-2-methylpyrimidine, B3PymPm, Liq and Al.

The following procedure describes the fabrication of the insulation device shown in Fig. 3a. For the no-insulation device, the PMMA layer process is skipped, whereas the same thermal evaporation steps apply.

An ITO-glass substrate (ITO sheet resistance, 10–15 Ω sq^−1^) was cleaned in acetone and isopropanol, followed by oxygen-plasma treatment at 200 W for 10 min (Plasmalab 80 Plus, Oxford Instruments). Microfiltered PEDOT:PSS solution was spin coated as a hole injection layer (Clevios P VP CH 4083, Heraeus), baked at 120 °C for 15 min. A nanostencil chip is attached on the substrate with alignment to the prepatterned ITO electrodes. For devices with an insulation layer, a thin PMMA layer (AR-P 672.045, Allresist) was spin coated and baked at 180 °C for 5 min. Oxygen-plasma etching was used to etch the PMMA layer through the nanostencil and expose the underlying PEDOT:PSS layer. Following organic layers were thermally evaporated through the nanostencil: TAPC (hole transport, 40 nm)/Ir(ppy)_3_:CBP (emission, 20 nm). Then, the nanostencil is detached from the substrate. The following layers were thermally evaporated through a stainless steel shadow mask: B3PymPm (electron transport, 50 nm)/Liq (electron injection, 2 nm)/Al (cathode, 70 nm).

### Nano-OLED morphological and topographical characterizations

SEM (Scios 2 DualBeam, Thermo Fisher Scientific) images were taken to characterize the morphology of the nanostructures and optimize the etching parameters for large-area uniformity. AFM (Park NX10, Park Systems) was used to acquire the high-resolution images of the nanostructures and their topographical characteristics. The measurements were done in the non-contact mode using a force modulation probe (PPP-FMR, Nanosensors). TEM (Talos F200X, Thermo Fisher Scientific) measurements were performed in the dark and bright-field modes to investigate the structural information of the cross-section of nano-OLED devices. The focused-ion-beam (Helios NanoLab 600i, Thermo Fisher Scientific) technique was used to prepare the lamella sample from the nano-OLED device for TEM measurement. A protective carbon layer was deposited on top of the nanopattern. Then, Ga liquid ion source was used to mill the area around the protection layer. EDS measurements were performed using the same instrument as the TEM but using a Super-X EDS detector.

### Device characterization

The current–voltage, luminance and EQE characteristics of nano-OLEDs are measured using two source measurement units (Keithley 2450, Tektronix) and a large-area (10 mm × 10 mm) calibrated Si photodiode (FDS1010-CAL, Thorlabs) placed in direct contact with the bottom surface of the device substrate to ensure underfilling of the detector and the collection of all photons in the forward direction^51^ (Supplementary Fig. 3a). The reference planar OLEDs (Extended Data Fig. 2) are measured using an absolute EQE measurement system (C9920-12, Hamamatsu Photonics) consisting of an integrating sphere, a photonic multichannel analyser (C10027-01, Hamamatsu Photonics) and a source measurement unit (Keithley 2450).

The EL spectra are measured with a spectroradiometer (PR 655 SpectraScan, Photo Research) or an imaging spectrograph (Kymera 328i, Andor).

### Optical characterization

A schematic of the custom-built optical characterization setup is displayed in Supplementary Fig. 3b,c. EML nanostructure arrays and nano-OLEDs are measured with an inverted microscope (Eclipse Ti2-U) equipped with a ×50 dry objective (TU Plan Fluor, NA = 0.8, working distance of 1.00 mm). For PL measurements, the samples are excited with wide-field illumination using a 400-nm LED source (pE-300, CoolLED). The emission is collected by the same objective and sent through a dichroic beamsplitter (LF-405/LP-B filter cube, Semrock) and to the exit port of the microscope, where an iris in the image plane is used to select the region of interest. An achromatic doublet (AC254-200-A, *f* = 200 mm, same as the microscope’s tube lens; Thorlabs) placed with the image plane in its front focal plane produces the desired *k*-space image onto its BFP where an imaging spectrograph (Kymera 328i, Andor) with its slit wide open relays the image to a scientific complementary metal–oxide–semiconductor camera (Zyla 4.2 PLUS, Andor). For imaging, a mirror is used instead of a zero-order grating to avoid aberrations. The same setup can image the real space by placing a *f* = 100 mm lens halfway between the image plane and the Fourier lens. For EL measurements, the devices are contacted from the top with tungsten probes (Signatone) connected to a source measurement unit (Keithley 2450, Tektronix). Angle-resolved PL, EL and reflectivity spectra are obtained by closing the spectrometer slit onto the *k*-space image and the image is dispersed by a grating (150 lines mm^−1^ blazed at 500 nm) in the direction orthogonal to the slit. The sample is rotated to ensure that the *k*_*y*_ axis of the patterns is parallel to the slit. A linear polarizer is inserted before the Fourier lens with its axis parallel or orthogonal to the spectrometer’s entrance slit to obtain the angle-dependent second Stokes parameter S_1_ and the polarized spectra.

### Optical simulations

Numerical simulations were performed using Lumerical (Ansys). The far-field radiation patterns and dispersions (Supplementary Fig. 26) from periodic structures were calculated using the rigorous coupled-wave approximation. The device unit cell was illuminated by a plane wave of wavelength *λ*, incident angle (*θ*, *ϕ*) and *s* or *p* polarization. The polar angle *θ* was varied from 0° up to 53°, corresponding to the objective’s NA, whereas the azimuthal angle from 0° to 360°. A small imaginary part (0.001i) was added to the refractive index of the EML to ensure the reciprocity principle could be used to estimate the emission into the far field^52,53^. All the other dielectric materials were considered lossless and dispersion was accounted for by using wavelength-dependent refractive index. The procedure yields the transmitted- and reflected-field intensities, which can be combined to calculate the resulting absorption by energy conservation. The final far-field radiation pattern or dispersion is obtained by integrating the absorption weighted by the PL or EL spectrum. The field profiles shown in Supplementary Fig. 5 are obtained from the same simulation using the finite-difference time-domain method with plane-wave illumination at normal incidence.

## Online content

Any methods, additional references, Nature Portfolio reporting summaries, source data, extended data, supplementary information, acknowledgements, peer review information; details of author contributions and competing interests; and statements of data and code availability are available at 10.1038/s41566-025-01785-z.

## Supplementary information

Supplementary Information Supplementary Discussions 1–3, Figs. 1–26 and Tables 1–10.

## Data Availability

All generated data and analyses that support this study are included within the Article and its Supplementary Information. The data for the figures in the main text are available via Zenodo at 10.5281/zenodo.17132301 (ref. ^54^). Additional data supporting the findings of this study are available from the corresponding authors upon reasonable request.
