# Supplementary material for: Scalable nanopatterning of organic light-emitting diodes beyond the diffraction limit
Source: Nat Photonics. 2025 Oct 31;20(1):31–9. doi: 10.1038/s41566-025-01785-z (PMC12774858; doi:10.1038/s41566-025-01785-z)
Supplement: Supplementary file 1 — Supplementary Discussions 1–3, Figs. 1–26 and Tables 1–10. [file 41566_2025_1785_MOESM1_ESM.pdf]

nature photonics

Supplementary information https://doi.org/10.1038/s41566-025-01785-z

Scalable nanopatterning of organic light-emitting diodes beyond the diffraction limit

In the format provided by the authors and unedited

Table of Contents

Supplementary discussion 1 | Simulation of Deposition Profile 3

Supplementary discussion 2 | Polarization response of organic nanorod meta-atoms........ 5

Supplementary discussion 3 | Methods for MD Simulations... 6

Supplementary Figure 1 | Nanostencil fabrication 8

Supplementary Figure 2 | Large area SEM images 9

Supplementary Figure 3 | Nano-OLED characterization 10

Supplementary Figure 4 | Polarization response of an organic nanorod metaatom 11

Supplementary Figure 5 | Electric field profiles for electroluminescent metasurfaces....... 12

Supplementary Figure 6 | Comparison between PL and EL angle-dependent spectra a-d . 13

Supplementary Figure 7 | Symmetry protected BIC formation in electroluminescent metasurfaces 14

Supplementary Figure 8 | Structure of the CBP (a) and Ir(ppy)3 (b) molecules................. 15

Supplementary Figure 9 | Simulation system for the 11 nm diameter droplet. 15

Supplementary Figure 10 | Estimation of the Stencil-Substrate Gap................................ 16

Supplementary Figure 11 | Gap estimation with a photospacer...................................... 17

Supplementary Figure 12 | Gap estimation without photospacer 18

Supplementary Figure 13 | Topographic profiles of organic semiconductor nanopatterns and fitting with parabolic function. 19

Supplementary Figure 14 | Modeling pattern formation of organic molecules deposited through a nanoaperture of SiNx membrane... 20

Supplementary Figure 15 | Calculated deposit profiles 21

Supplementary Figure 16 | Comparison between experimental AFM-measured and computational lithography (CL) calculated profiles. 22

Supplementary Figure 17 | Schematic of nanostencil shadowing 23

Supplementary Figure 18 | Topographical homogeneity for bare EML nanodisks deposited through nanostencils. 24

Supplementary Figure 19 | Emission non-uniformity in non-optimized devices. 25

Supplementary Figure 20 | Improved EL uniformity for our newly fabricated nano-OLED devices (nanodisk array at 10,000 ppi, diameter of 500 nm and periodicity of 2.5 µm)..... 26

Supplementary Figure 21 | Improved EL uniformity for our newly fabricated nano-OLED devices (nanodisk array at 25,000 ppi, diameter of 200 nm and periodicity of 1 µm)........ 27

Supplementary Figure 22 | Size dependence of nano-OLED performance......................... 28

Supplementary Figure 23 | Schematic diagram illustrating the layout of a full nano-OLED device. *29*

Supplementary Figure 24 | Simulations of outcoupling efficiency from nano-OLEDs *30*

Supplementary Figure 25 | Nano-OLED operational lifetime. *30*

Supplementary Figure 26 | RCWA dispersion of nano-OLEDs. *31*

Force-field parameters for CBP *32*

Supplementary Table 1 | CHARMM bonded parameters from the SwissParam server. **32**

Supplementary Table 2 | CHARMM angle parameters from the SwissParam server. **32**

Supplementary Table 3 | CHARMM dihedral parameters from the SwissParam server **34**

Supplementary Table 4 | CHARMM improper parameters from the SwissParam server. **35**

Supplementary Table 5 | CHARMM Lennard-Jones parameters from the SwissParam server **35**

Force-field parameters for Ir(ppy)$_3$ *36*

Supplementary Table 6 | Bonded parameters developed using ffTK **36**

Supplementary Table 7 |. Angle parameters developed using ffTK **36**

Supplementary Table 8 | Lennard-Jones parameters obtained from ffTK. **37**

Supplementary Table 9 | Statistical analysis for gap distances at different areas. **38**

Supplementary Table 10 | Reference OLED performance and effects of PEDOT:PSS over-etching. **38**

Supplementary discussion 1 | Simulation of Deposition Profile

We consider the molecular flux generated from an evaporation source, which has an angular distribution as a function of the projection angle, $\varphi \geq 0$ (**Supplementary Fig. S14a**; see above). As illustrated in **Fig S15b**, when the projection angle is greater than the critical angle, $\varphi_c = \tan^{-1}(L/\delta)$, the flux is blocked by the SiNx sidewall, where L is the nanoaperture width and δ is the membrane thickness. Clearly, the higher aspect ratio of the aperture (δ/L), the smaller φ_c. Accordingly, upon evaporation, the projection of molecular flux passing through a nanoaperture results in a different shape from the aperture design. This phenomenon has been extensively analyzed in another recent publication from our group[1]. We have used the same mathematical framework of computational lithography (CL) to model the deposited profiles with the following assumptions:

- We assume that the sidewall of the nanoaperture has a vertical structure.
- The vapor plume from the evaporation source consists of molecular beams of various angles and is characterized by the angular distribution function $p(\varphi)$, where θ is an azimuthal angle and φ is a polar angle (see **Fig. S14a**). The parametric hemisphere that characterizes the trajectory of the vapor plume is illustrated in **Supplementary Fig. S17e** attached below.
- We assume perfect alignment between source and the substrate, so that the molecular plume has azimuthal symmetry. We model $p(\varphi)$ using a powered cosine distribution, $p(\varphi)=\cos^n(\varphi)$, where n is a positive real number characterizing the sharpness of the emission profile[2].
- We neglect surface diffusion of the organic molecules.

Accordingly, the deposition on a flat substrate surface is governed by the intrinsic angular distribution of molecular flux, $F(\varphi)$, given by:

$$F(\varphi)=F_0 p(\varphi) \qquad \text{(S1)}$$

where F_0 is the flux at the normal incidence, $\varphi=0$, and $p(\varphi)$ is the angular distribution function of the vapor plume, as shown in **Supplementary Fig. S14a**. The deposited film thickness without the stencil, H_0, is therefore given by:

$$H_0(t)=t \cdot \int_{-\frac{\pi}{2}}^{\frac{\pi}{2}} F(\varphi)d\varphi \qquad \text{(S2)}$$

where t is the deposition time. Accordingly, the following two effects determine the deposit profiles:

first, because a given molecular particle can only land on the substrate when its trajectory passes through the nanoapertures, for a nanoaperture with opening width L and membrane thickness δ, the deposition is blocked for $\varphi > \varphi_c$. This is what we describe as, the *self-shadowing effect* (**Supplementary Fig S14b**).

Next, when the deposition proceeds, the accumulated material on the stencil's top and side walls reduces the effective opening, namely, the *clogging effect*. We model this effect by allowing the membrane and opening width to vary with time by $\Delta\delta$ and ΔL, respectively (**Fig. S14c**), which are approximated to be proportional to the nominal thickness, $H_0(t)$, following $\Delta\delta(t)=\lambda_\delta H_0(t)$ and $\Delta L(t)=\lambda_L H_0(t)$. Accordingly, assuming the center of the 1D slit is located at $x=0$, the material flux at given location x on the substrate is given by:

$$\frac{\partial H(t)}{\partial x} = \int_{-\varphi_c}^{\varphi_c} \Theta(x-x_l)\Theta(x_r-x)F_0 p(\varphi)d\varphi \qquad \text{(S3)}$$

where Θ is the Heaviside step function, x_l and x_r are the left and right boundaries of the deposited area, respectively.

As shown in **Supplementary Figure S15**, varying L significantly alters the shape of the deposited patterns, from a Gaussian-like distribution at L=50 nm (**Figure S15a**) to a trapezoidal profile at $L = 1500$ nm (**Figure S15c**). The self-shadowing effect, as demonstrated in Supplementary Eq. 3, strongly suppresses deposition height when $L \approx \delta$, consistent with the experimental trends observed in Fig. 2c and 2d.

To further assess the impact of material accumulation during evaporation, we compared simulations with and without the clogging effect, using a nominal deposition thickness $H_0 = 100$ nm and buildup parameters $\lambda_\delta = 1.0$ and $\lambda_L = 0.5$. As expected, the inclusion of the clogging effect further reduced the deposition thickness by almost half in L=50 nm case. In contrast, for wider stencils (L = 500 nm and L = 1500 nm), the effect of clogging on the overall profile was almost negligible.

Using Supplementary Eq. 3, **Supplementary Fig. S16** compares the AFM-measured and CL-calculated deposit profiles and the maximum height H_{max}. Overall, the trend found in our experimental data showed in **Figs. 2c** and **2d** is nicely captured and it becomes evident that the pattern broadening and height reduction comes mostly from the air gap between the stencil and the substrate, and for small nanoaperture partially from material build-up.

Supplementary discussion 2 | Polarization response of organic nanorod meta-atoms

To understand the polarization response of the electroluminescent metasurfaces based on 1D linear arrays of organic nanorods we start by considering light emission from a single nanostructure (see Fig. S4a). The polarization state of light, even if its partial, can be completely described by the four Stokes parameters (S_0, S_1, S_2, S_3). These quantities are readily measurable through a total of six intensity measurements projected on specific polarization basis: horizontal, vertical, diagonal, anti-diagonal, left-handed and right-handed circular. Specifically, these quantities have the following physical interpretation: S_0 measures the total field intensity, S_1/S_0 quantifies whether the light is preferentially polarized along the vertical(parallel)/horizontal(perpendicular) basis, S_2/S_0 quantifies the polarization in the diagonal/anti-diagonal basis and S_3/S_0 specifies the degree of circular polarization. Due to the normalization by the total intensity S_0, the Stokes parameters range between -1 and 1, where the extrema characterize complete polarization along one of the two appropriate basis vectors. In our case, we are only interested in the degree of linear polarization with respect to the basis parallel (k_x) and perpendicular (k_y) to the nanorods and therefore to the S_1/S_0 parameter at different angles.

The dielectric contrast between the nanorod and its surrounding medium leads to the renormalization of the local electric field within the structure[3]. As a consequence, the emission intensity for a certain polarization direction is reduced (screened) depending on its angle with the nanostructure axes. In our case, we assume isotropic emission within the wire due to the random orientation of the organic molecules. For simplicity, elongated structures, such as nanorods and nanowires, are modelled as prolate ellipsoids and Figure S4b shows the screening factors $D_{\parallel}$ and $D_{\perp}$ for dipoles along the long and short axis as a function of the aspect ratio b/c of the ellipsoid. Since our nanorods have an aspect ratio of 0.02 (dashed line in Fig. S4b) they can be accurately approximated as infinite wires ($\frac{b}{c} \rightarrow 0$). In this limit, there is no reduction of transitions polarized along the long axis ($D_{\parallel} = 1$) while emission polarized along the short axis is screened as

$$D_{\perp} = \left(\frac{2}{1+\epsilon}\right)^2 (S_1) \qquad \text{(S4)}$$

Accordingly the maximum degree of linear polarization for emission perpendicular to the substrate is given by $S_1/S_0 = (1 - D_{\perp})/(1 + D_{\perp})$ [4], which for our structure in air amounts to

a value of ≈ 0.6. For light emitted in a different direction we refer to the scheme in Fig. S4a and consider first two limiting cases. First, for directions in a plane orthogonal to the long axis of the wire (φ = 0°), the s-polarization is unaffected as it is always parallel to the long axis, while p-polarization is reduced by a factor $D_{\perp}$, independent of θ for circular cross-section. On the other hand, for directions in the plane parallel to the long axis (φ = 90°), the s-polarized emission always lies along the short axis and it is screened by $D_{\perp}$, while the screening of the p-polarized component depends on the angle θ and it is given by a factor $cos^2\theta + D_{\perp}sin^2\theta$. Indeed, for θ = 0° the p-polarization is parallel to the long axis and it's unaffected but as the angle changes the screening increases due to the component along the short axis. This is true in the local s and p polarization basis, but in order to calculate S_1, we need to consider the transformation to the vertical (parallel)/horizontal (perpendicular) basis, as the spherical waves are collimated by the objective lens. We notice for example that the parallel polarization component derives from the s-polarization for φ = 0° but from the p-polarized component at φ = 90°. Accordingly, S_1/S_0 is given by

$$S_1/S_0\,(\theta, 0°) = \frac{1 - D_{\perp}}{1 + D_{\perp}}$$

$$S_1/S_0\,(\theta, 90°) = cos^2\theta + D_{\perp} \qquad \text{(S5)}$$

$$S_1/S_0\,(\theta, \phi) = S_1/S_0\,(\theta, 0°)cos^2\phi + S_1/S_0\,(\theta, 90°)sin^2\phi$$

and it is plotted in Fig. S4c. As a result, the dielectric nanorod is a promising metaatom geometry for the control of linear polarization as it exhibits polarized emission along its axis in all directions with the maximum at θ = 0° and a slight reduction at off-axis angles along the θ = 90° direction. We believe that the polarization response of our organic nanorod arrays on glass is dominated by that of the single meta-atom as demonstrated by the weak dependence of S_1/S_0 images to the array spacing (see Fig. 6c,f and Extended Data Fig. 7b,e).

Supplementary discussion 3 | Methods for MD Simulations

We performed large-scale MD simulations to obtain detailed insights into the structure of the CBP and $Ir(ppy)_3$ molecules in the nano-OLED device. We generated the structures of the CBP and $Ir(ppy)_3$ molecules using Avogadro[5] and VMD[6]. The chemical composition of the CBP and $Ir(ppy)_3$ molecules are $C_{36}H_{24}N_2$ and $C_{33}H_{24}IrN_3$, respectively as shown in Fig. S8. Using these molecules, we constructed an organic semiconductor droplet which contains 8 wt. % $Ir(ppy)_3$ molecules. Two different droplet sizes were considered with diameters of 9 nm

and 11 nm (these values are obtained after equilibration). The 9 nm diameter droplet contained 311 CBP molecules and 20 $Ir(ppy)_3$ molecules, while the 11 nm diameter droplet consisted of 622 CBP molecules and 40 $Ir(ppy)_3$ molecules.

Using these droplets, simulation systems were constructed using Packmol[7] and VMD[6]. The simulation system for the 11 nm diameter droplet is shown in Fig. S9. Three graphene sheets stacked one over another in the AB stacking configuration were considered to form the model graphite substrate. Each of the graphene sheets contained 20,680 carbon atoms. All MD simulations reported in this study were performed in NAMD 2.14[8] with the CHARMM[9] force field. Force-field parameters for CBP molecules and graphene were obtained from the SwissParam server[10] while for $Ir(ppy)_3$ molecules, the Force Field Toolkit (ffTK 2.1)[11] was used to generate the force-field parameters. The force-field parameters are outlined in Tables S1-S8. Van der Waals interactions and exchange repulsion were modeled using a 6-12 Lennard-Jones potential with a cut-off distance of 12.0 Å. For computing the long-range electrostatic interactions, the particle mesh Ewald (PME)[12] method was employed. The simulation systems were first subjected to energy minimization and subsequently, MD simulations were performed at a constant temperature of 300 K in the canonical (NVT) ensemble. The temperature of the system was controlled using the Langevin thermostat[13] with a damping factor of 5 ps^{-1}. The graphene substrate was fixed in space during the simulations. A vacuum of 100 Å was applied on both sides of the simulation system along the *z* direction (leading to a total vacuum of 200 Å). Periodic boundary conditions were applied along all directions. The simulations were performed for 100 ns and data was recorded every 1 ps. For the steered MD simulations in a freestanding nano-OLED, the $Ir(ppy)_3$ molecule was pulled at a rate of 1 Å/ns with a force constant of 5 kcal/mol $Å^2$. Note that the calculated ΔA (Fig. 2f) is rather qualitative, due to the use of a fast pulling rate for the $Ir(ppy)_3$ molecule.

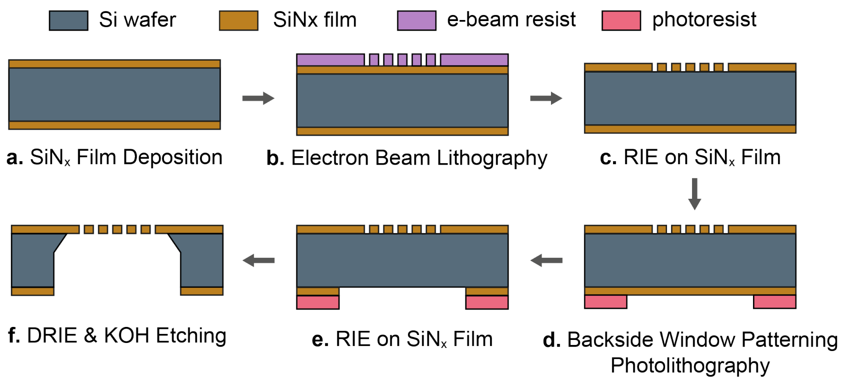

Supplementary Figure 1 | Nanostencil fabrication Schematic illustration of the nanostencil fabrication process. **a,** uniform SiN_x film is deposite on double-side-polished Si wafer using LPCVD. **b,** Nanopattern is defines on E-beam resist layer using e-beam lithography. **c,** RIE is used to etch the underlying SiN_x film to transfer the nanopattern from the resist layer. E-beam resist is then removed. **d,** Nanopattern area is defined on photoresist layer using photolithography. **e,** RIE used to etch the part of SiN_x film on the bottom side defined by photoresist. **f,** Si wafer on the window area is fully etched using the combination of DRIE (87%) and KOH wet etching (13%).

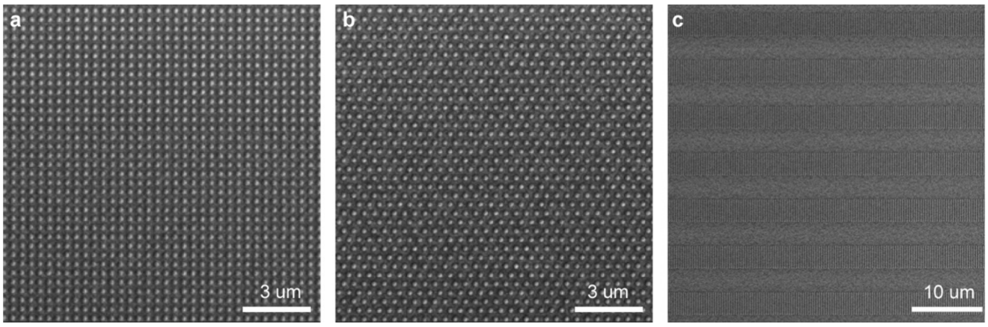

Supplementary Figure 2 | Large area SEM images a-c Low magnification SEM images of 2D square, 2D hexagonal and 1D linear arrays of $Ir(ppy)_3$:CBP nanostructures displaying uniformity across a large area.

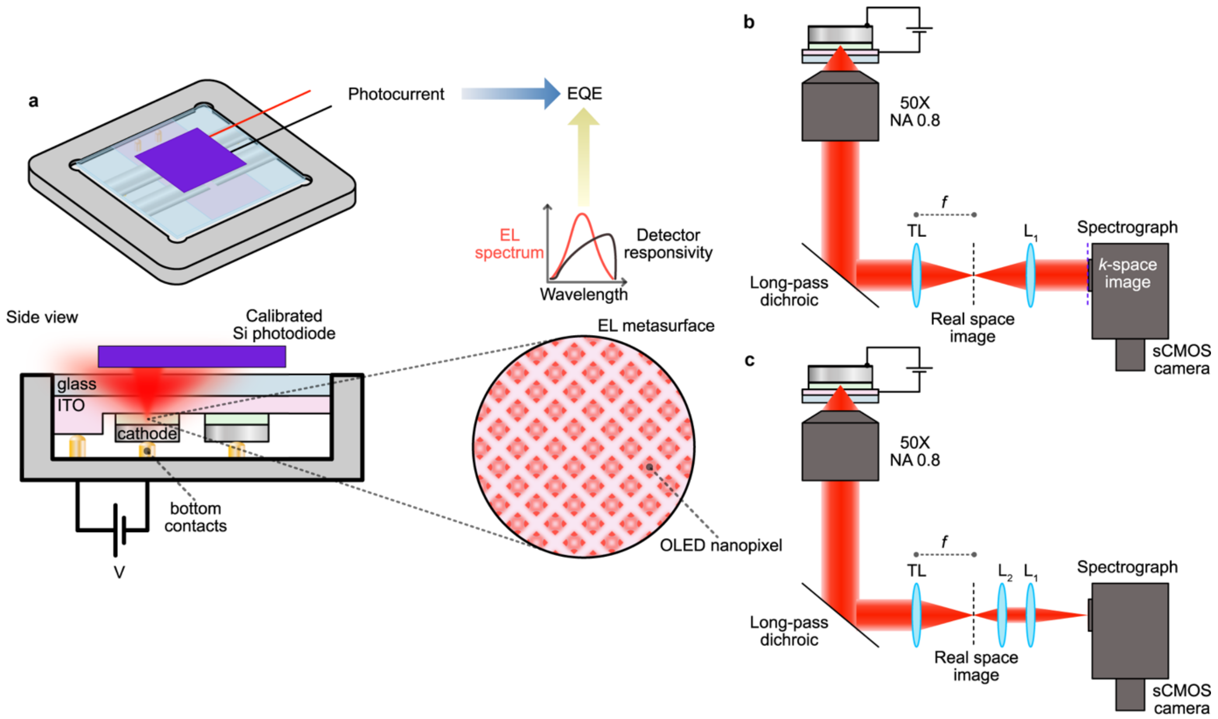

Supplementary Figure 3 | Nano-OLED characterization a-c Schematic diagrams of the Nano-OLED characterization procedures used in the main text. Respectively, EQE measurement using calibrated Si photodiode and BFP imaging setup in k-space **(b)** and real-space (**c)** mode.

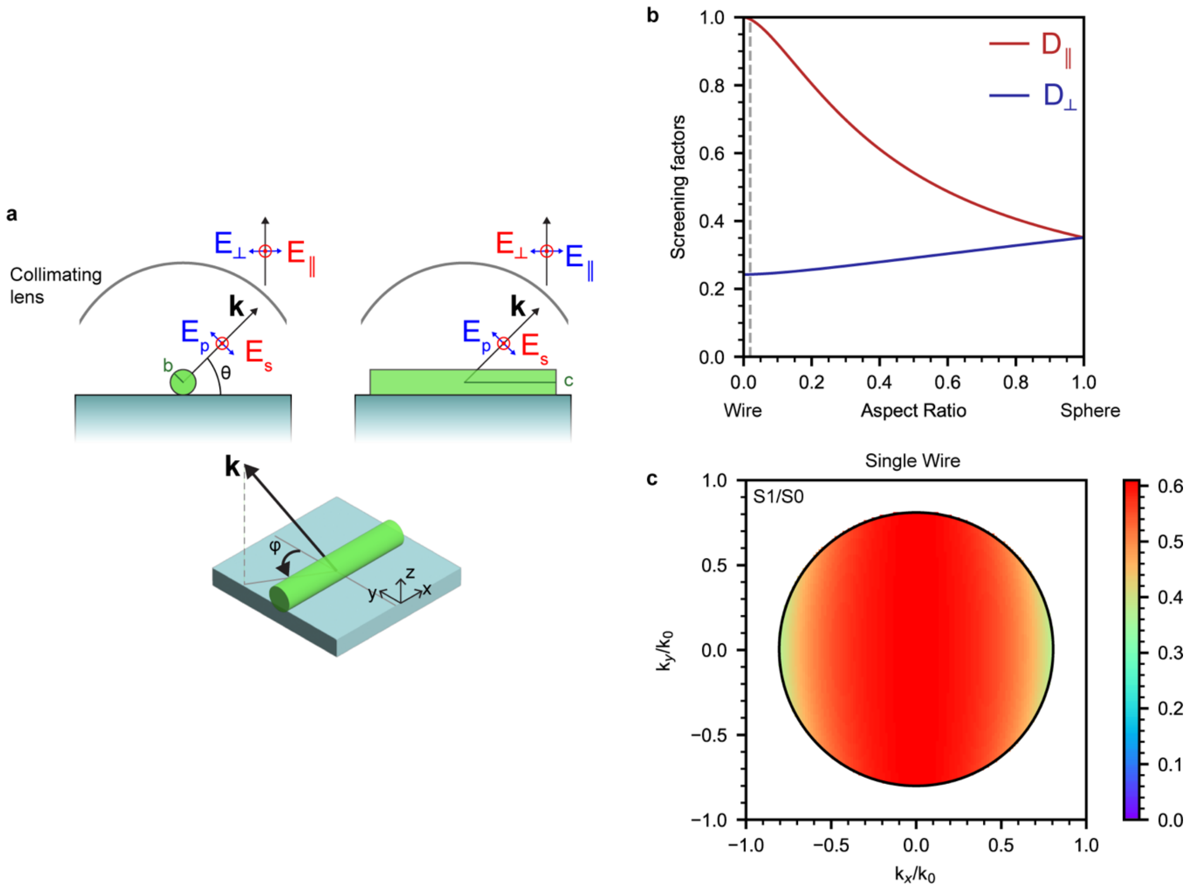

Supplementary Figure 4 | Polarization response of an organic nanorod metaatom a Schematic diagram of the geometry of light propagation from the nanorod in different directions. **b,** Screening factors as a function of aspect ratio b/c for a prolate ellipsoid with the experimental dielectric contrast. The dashed line correspond to the experimentally investigated structures. **c,** Calculation of S1/S0 for a single nanorod

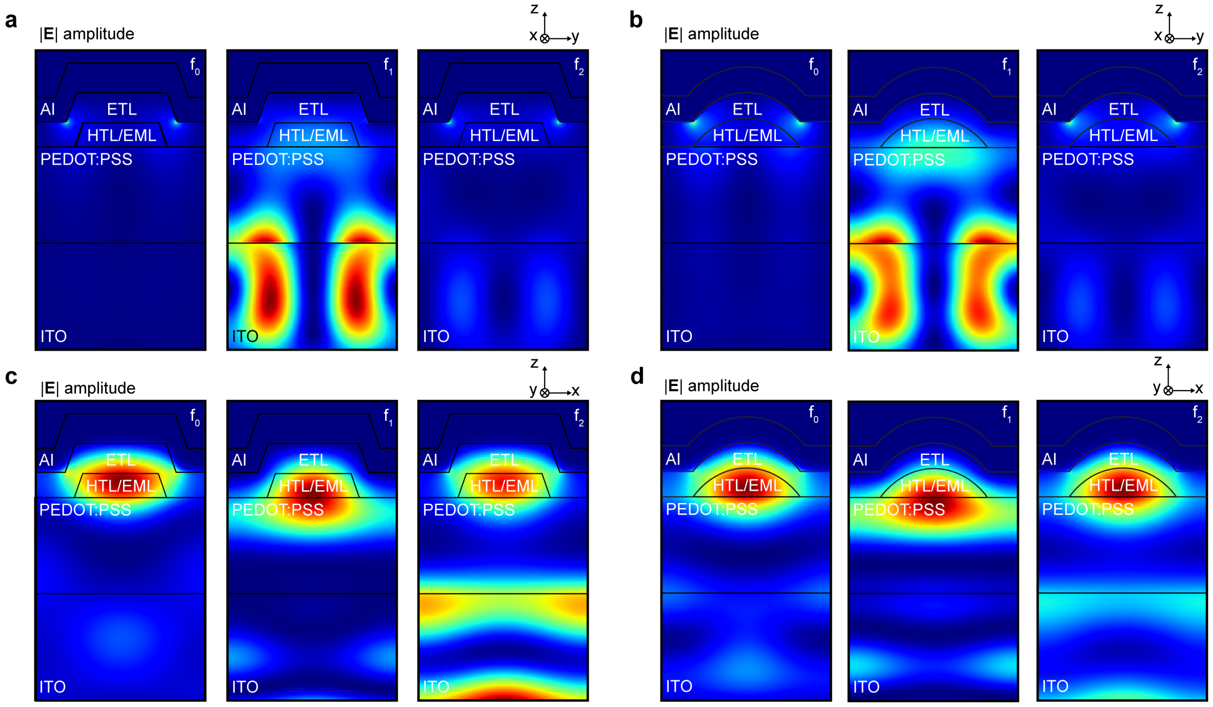

Supplementary Figure 5 | Electric field profiles for electroluminescent metasurfaces. a, Simulated electric field profile for the resonant modes highlighted in Fig. S26 at the Γ point of the structure in Fig. 4a. a, b The simulated electric field amplitude in the ZY-plane for the trapezoidal (a) and parabolic (b) EML topographic profiles. c, d The simulated electric field amplitude in the ZX-plane for the trapezoidal (c) and parabolic (d) EML topographic profiles.

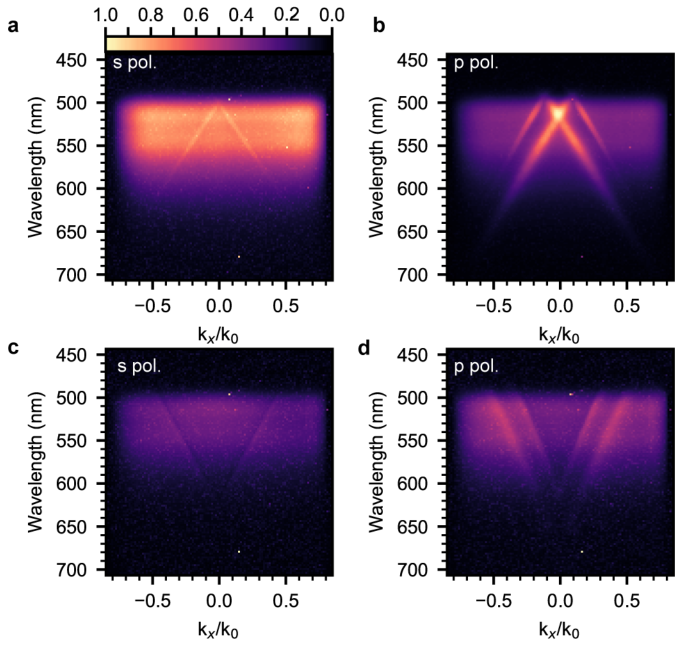

Supplementary Figure 6 | Comparison between PL and EL angle-dependent spectra a-d Representative angle-dependent EL spectra for the structures in Fig. 4 and Extended Data Fig. 4. All spectra were taken at a constant bias of 6V.

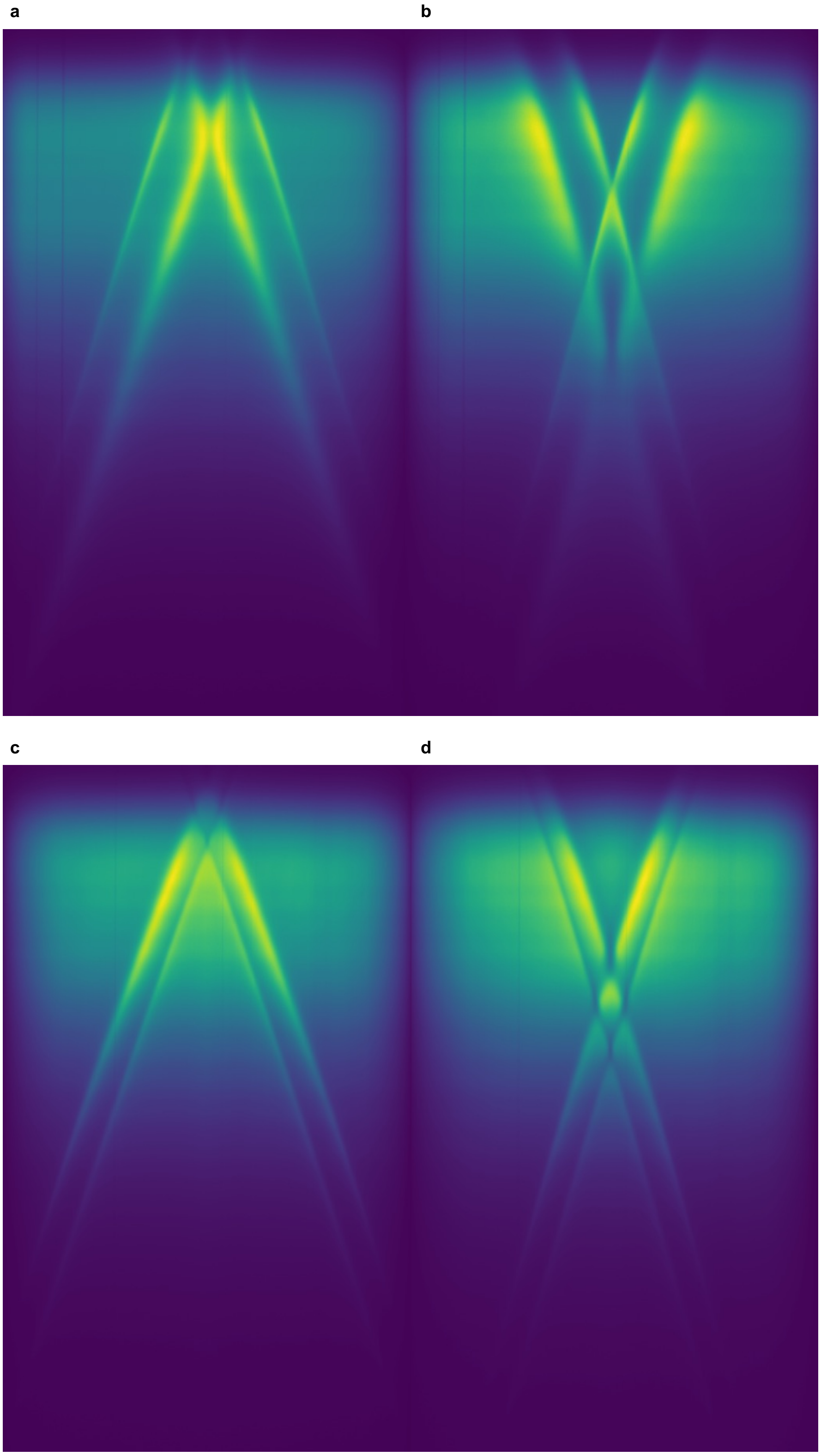

Supplementary Figure 7 | Symmetry protected BIC formation in electroluminescent metasurfaces a-d Raw data for the p-pol. angle dependent spectra in Extended Figs. 4, 7c and 8f,g showing more clearly the decrease of PL emission at the Γ point due to the formation of a symmetry protected quasi-BIC of the lowest plasmonic TM mode.

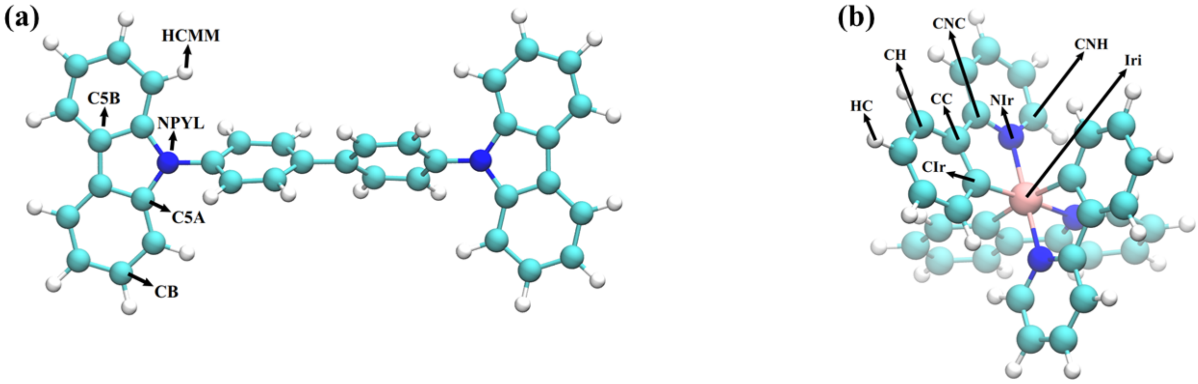

Supplementary Figure 8 | Structure of the CBP (a) and Ir(ppy)3 (b) molecules. Hydrogen atoms are in white, carbon atoms are in cyan, nitrogen atoms are in blue, and the iridium atom is in pink. The atom types of the molecules are also represented.

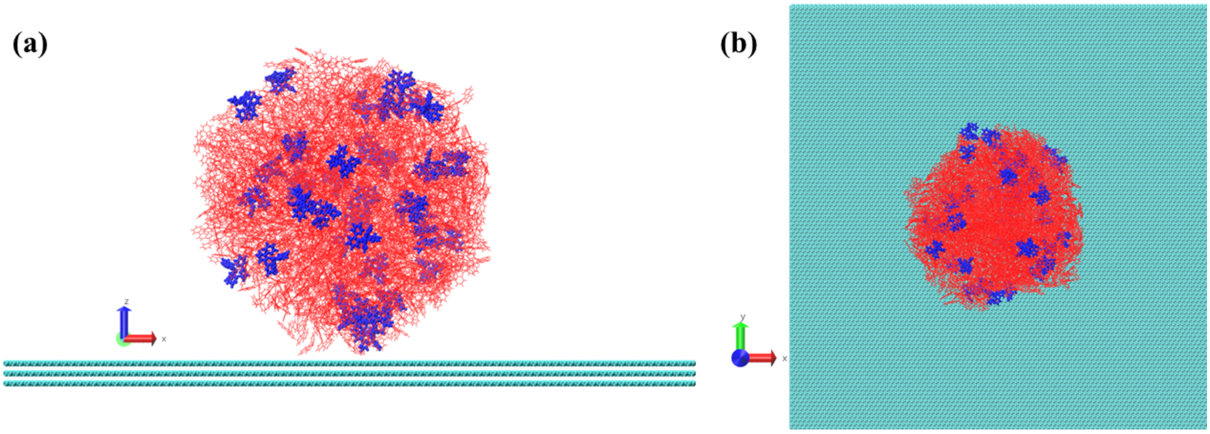

Supplementary Figure 9 | Simulation system for the 11 nm diameter droplet. a, Front view. **b,** Top view. CBP molecules are in red, Ir(ppy)$_3$ molecules are in blue, and carbon atoms are in cyan.

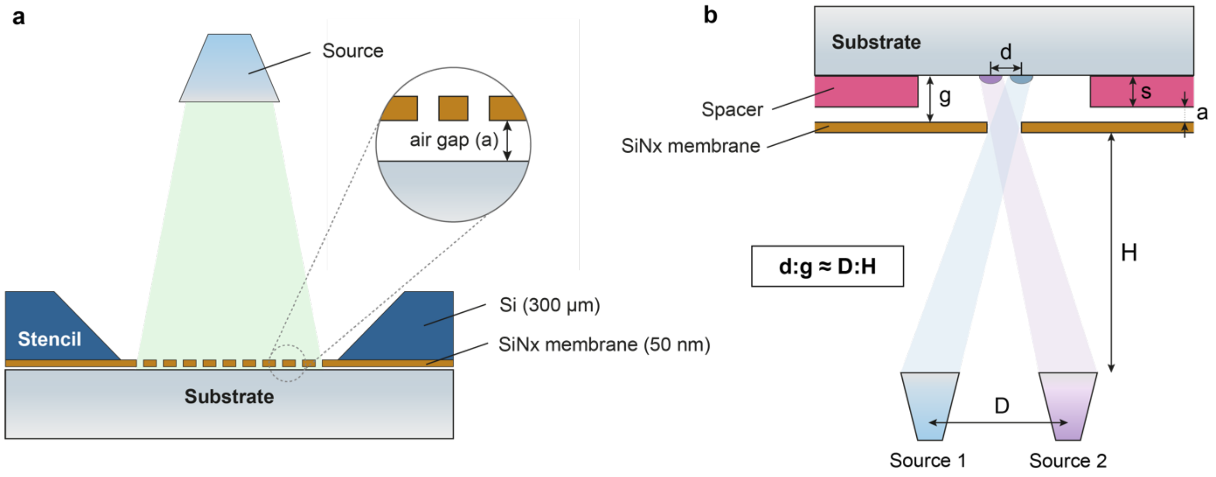

Supplementary Figure 10 | Estimation of the Stencil-Substrate Gap. a, Schematic diagram illustrating a nanostencil chip attached to a substrate before the evaporation of organic materials. The SiNx membrane faces the substrate to minimize the stencil-substrate gap (a). **b,** Schematic illustration of the experiment used to estimate the height of the air gap, g. Two organic materials were evaporated from two different sources of known position. Due to the existence of the air gap, the distance between the two sources, D, results in a displacement between the material deposits landing on the substrate, d, following $d: g \approx D: H$, where H is the vertical height from the source surface to the substrate.

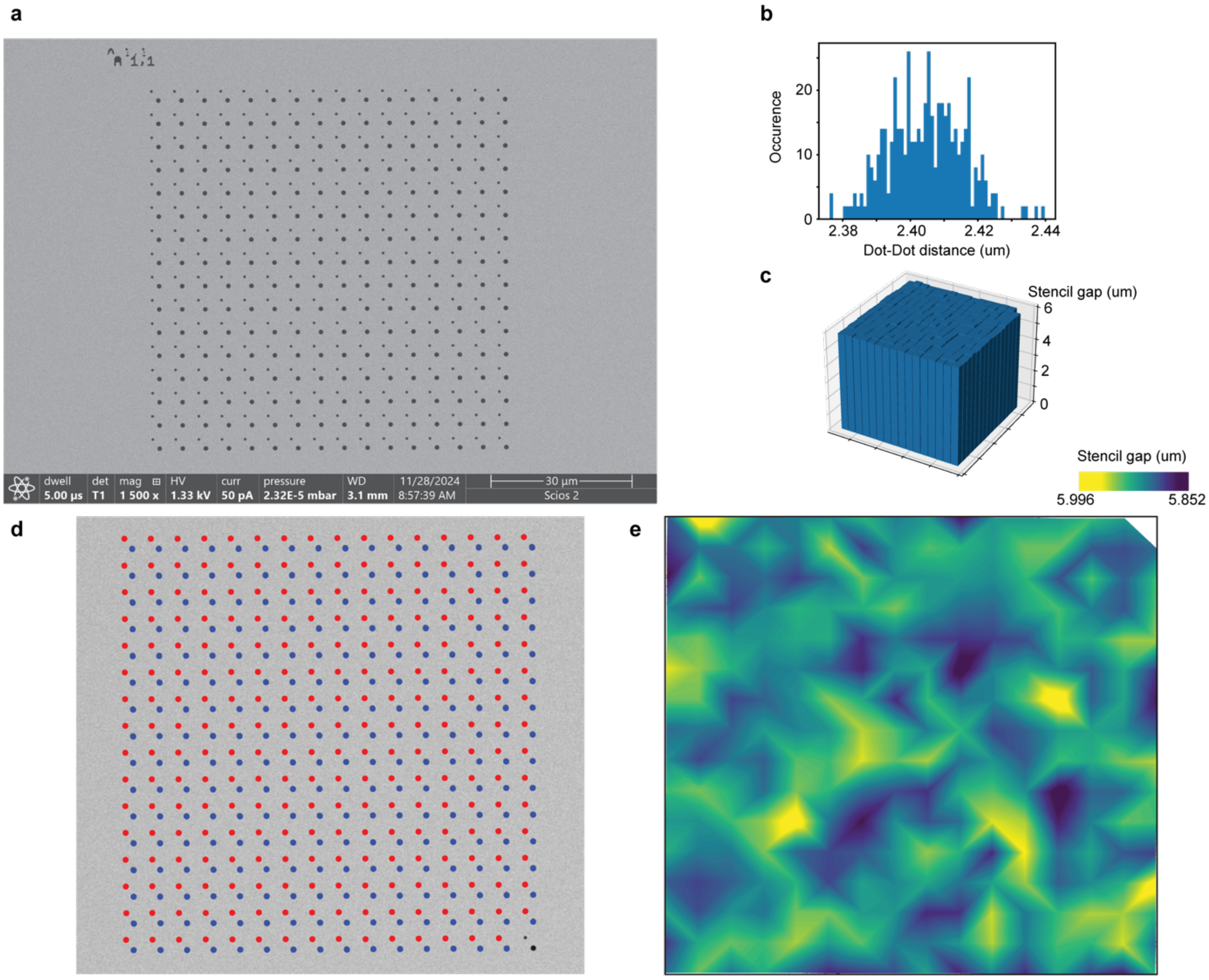

Supplementary Figure 11 | Gap estimation with a photospacer. (**a)** Representative SEM image of an area used for the estimation of the stencil-substrate gap. A photospacer of thickness 5.19 um is sandwiched between the stencil and substrate to increase the distance between the dots. **(b)** Histogram and (**c**) spatial distribution of the dot-dot distances extracted by automatic segmentation of the SEM image. **(d)** The dot deposits resulting from the centered (red) and off-centered (blue) evaporation sources. (e) The spatial contour map of the extracted stencil-substrate gaps.

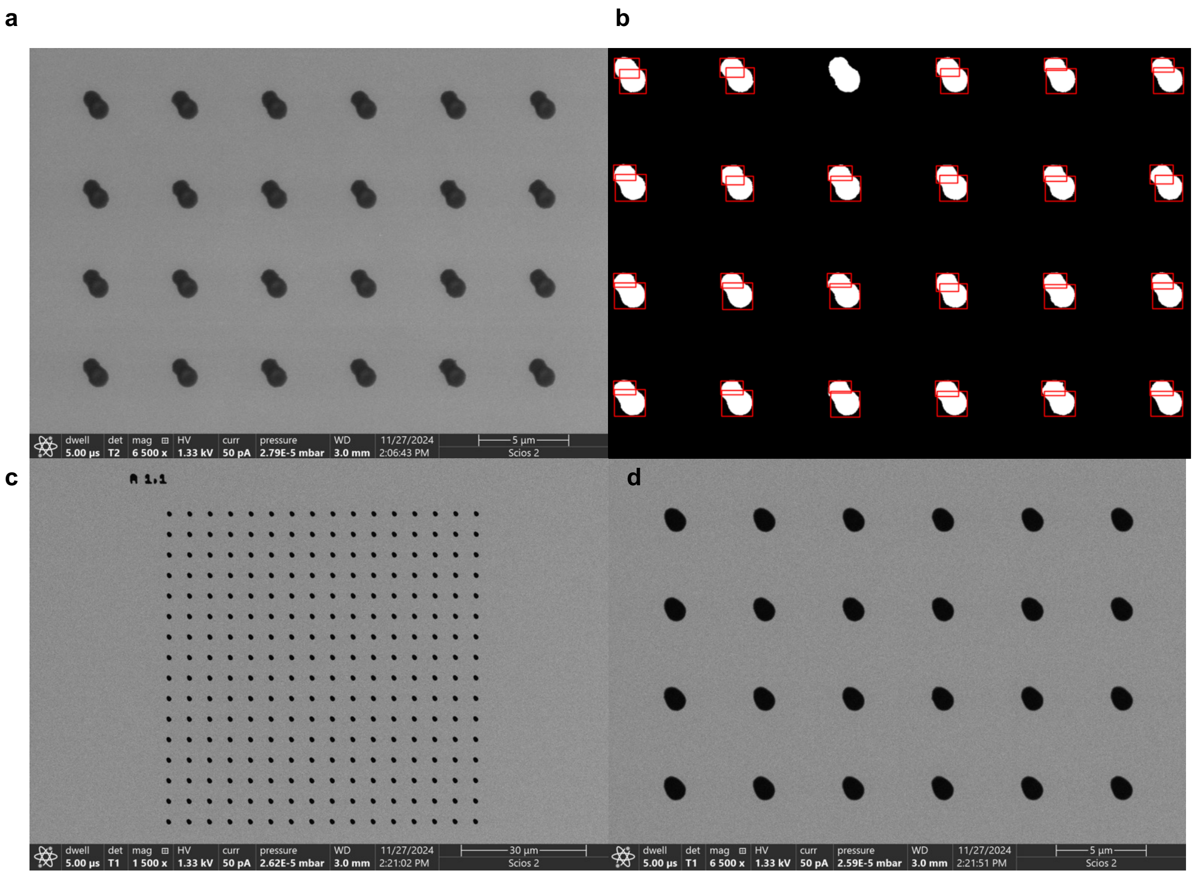

Supplementary Figure 12 | Gap estimation without photospacer (**a,c,d)** SEM images of two representative samples used for the estimation of the stencil-substrate air gap. Note that here there is no photospacer between stencil and substrate; the stencil is directly in contact with the substrate. **(b)** Example of automatic segmentation of overlapping patterns in the SEM image.

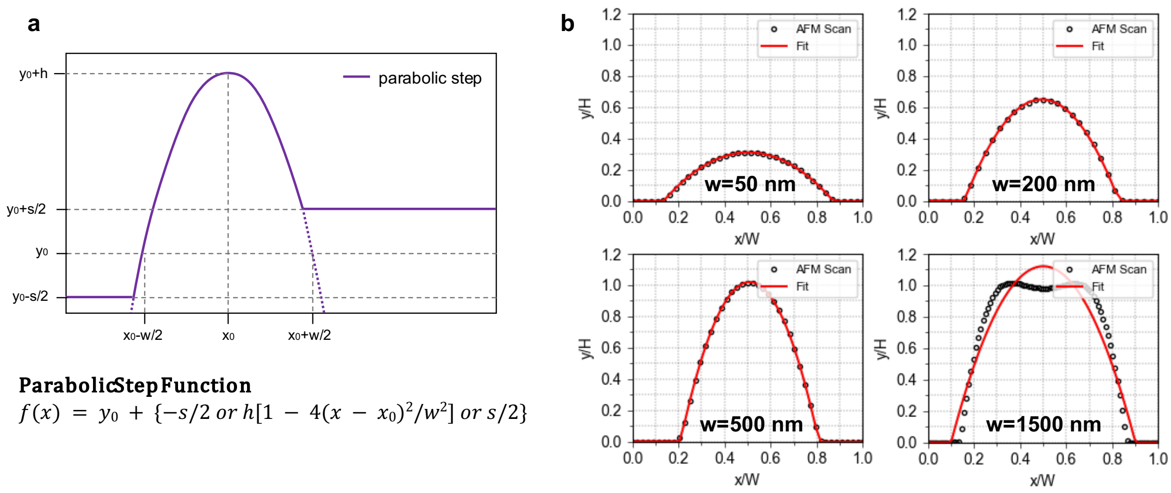

Supplementary Figure 13 | Topographic profiles of organic semiconductor nanopatterns and fitting with parabolic function. a, The parabolic step function used to fit the profiles, having 5 parameters, x_0, y_0, h, w, and s. **b,** Comparison of the AFM-scan profiles (circles) and their fittings (red curves). Plots represent normalized height as a function of dimensionless position, where H is the target height and W is the scan length.

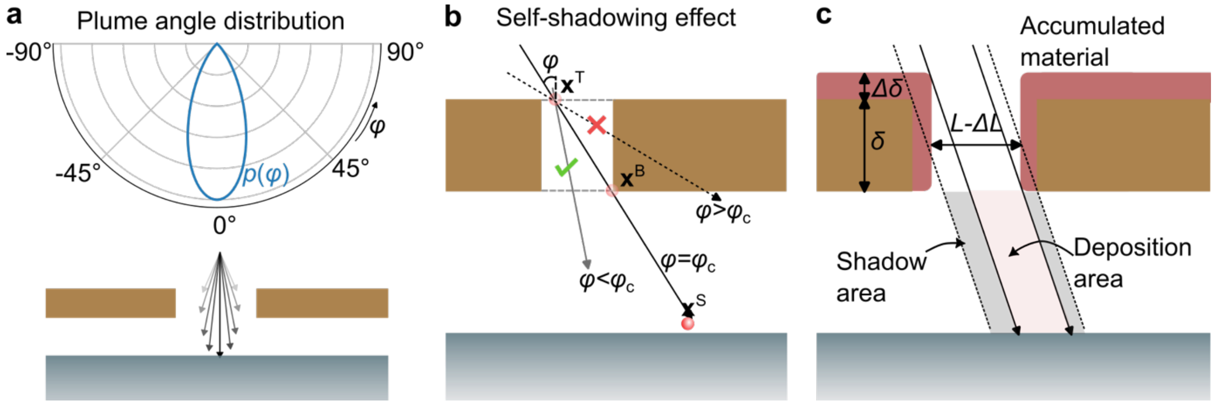

Supplementary Figure 14 | Modeling pattern formation of organic molecules deposited through a nanoaperture of SiNx membrane. a. Angular distribution function for the molecular flux coming from an evaporation source, namely the plume, $p(\varphi)$, as a function of the projection angle φ. The arrows represent the trajectories of molecular beams. **b**. Schematic diagram illustrating the self-shadowing effect. The geometry of the nanoaperture corresponds to a critical deposition angle φ_c, where no deposition occurs when $|\varphi|>\varphi_c$. **c.** Schematic illustration of the clogging effect due to the accumulation of material deposited on the top and sidewalls of the stencil membrane. This buildup increases the area of shadowed regions (gray), which reduces the effective deposition area (pink).

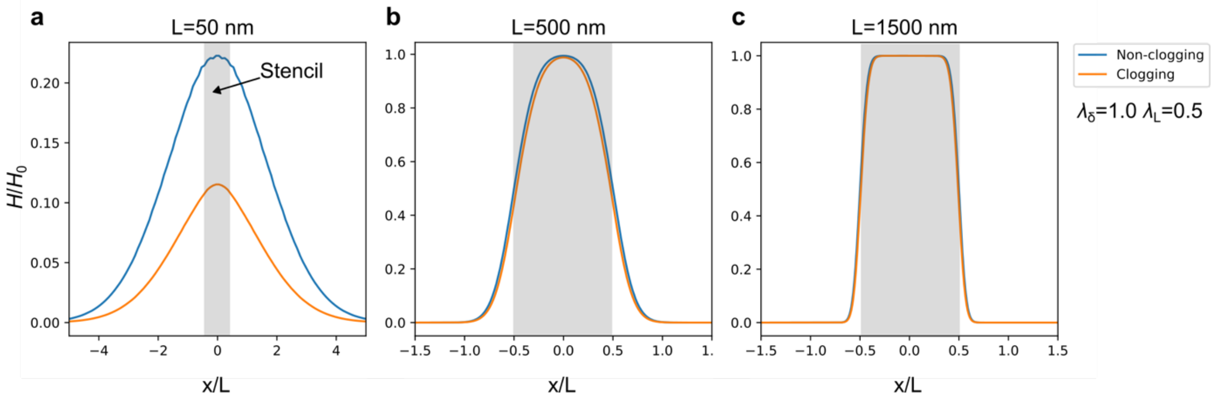

Supplementary Figure 15 | Calculated deposit profiles Calculated deposit profiles considering various nanoaperture openings, showing the normalized height profiles H/H_0 as a function of the dimensionless coordinate x/L, for **a**. $L = 50$ nm, **b**. $L = 500$ nm and **c**. $L = 1500$ nm slits, with (orange curves) and without (blue curves) taking into account the clogging effect. The nominal thickness H_0 is 100 nm and the clogging effect parameters are λ_δ = 1.0 and λ_L = 0.5. The nanoaperture opening corresponds to the gray area.

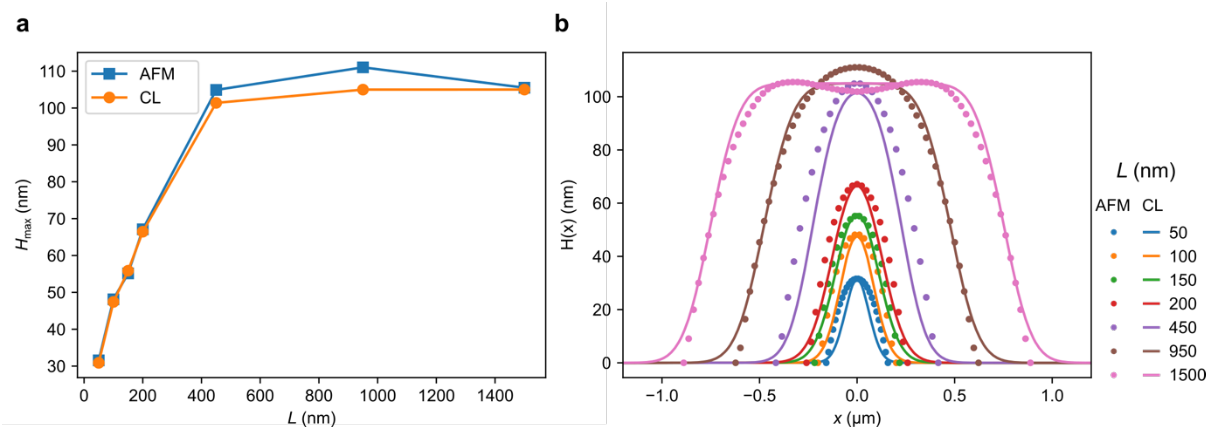

Supplementary Figure 16 | Comparison between experimental AFM-measured and computational lithography (CL) calculated profiles. a. Maximum height H_{max} extracted from the AFM measurements and simulations as a function of slit width L. **b.** Height profiles $H(x)$ from AFM measurements (dots) and CL simulations (lines) as functions of location x on the substrate. The slits are centered at x=0.

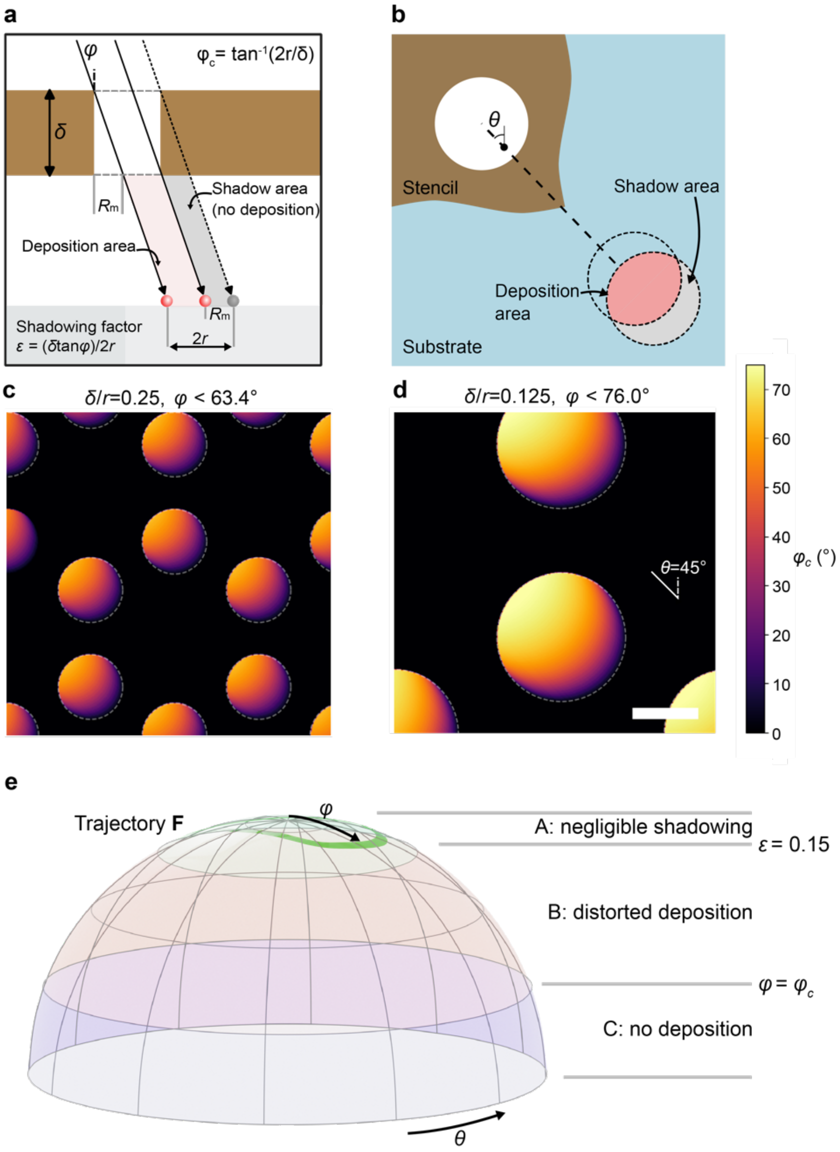

Supplementary Figure 17 | Schematic of nanostencil shadowing. **a,b.** the side and top views of the shadow cast by the stencil wall of the nanoapertures. The gray regions indicate areas where deposition is blocked by the membrane sidewall. The shadowing factor ε quantifies the fraction of the projected pattern obstructed by the shadow. **c,d.** Distribution of the critical deposition angle φ_c for the circular nanoapertures at incident azimuthal angle θ = 45°, for different aspect ratios δ/r. Deposition is completely blocked beyond the critical deposition angle, $\varphi_c = \tan^{-1}(2r/\delta)$. **e.** Operational regimes of MBHL. The parametric hemisphere is divided into 3 regimes based on the deposition angle φ. All figures are adapted from ref 1 with permission.

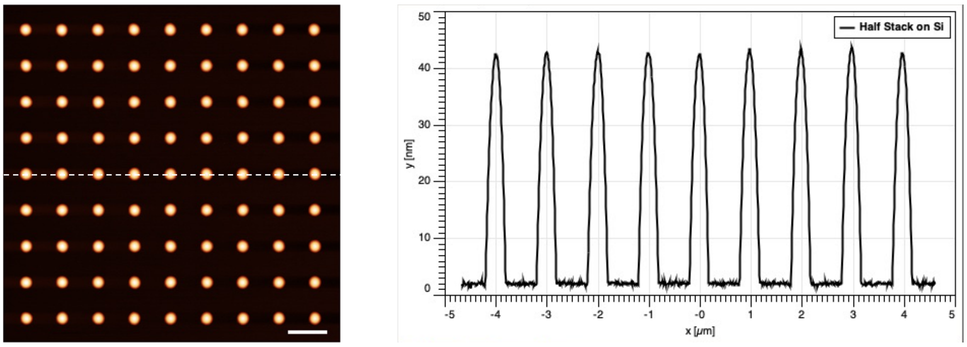

Supplementary Figure 18 | Topographical homogeneity for bare EML nanodisks deposited through nanostencils. The AFM image (left; scale bar: 1μm) and the extracted height profile (right) along the white dashed line suggest very high degree of profile uniformity for the deposited nanodisks on ultraflat silicon chip.

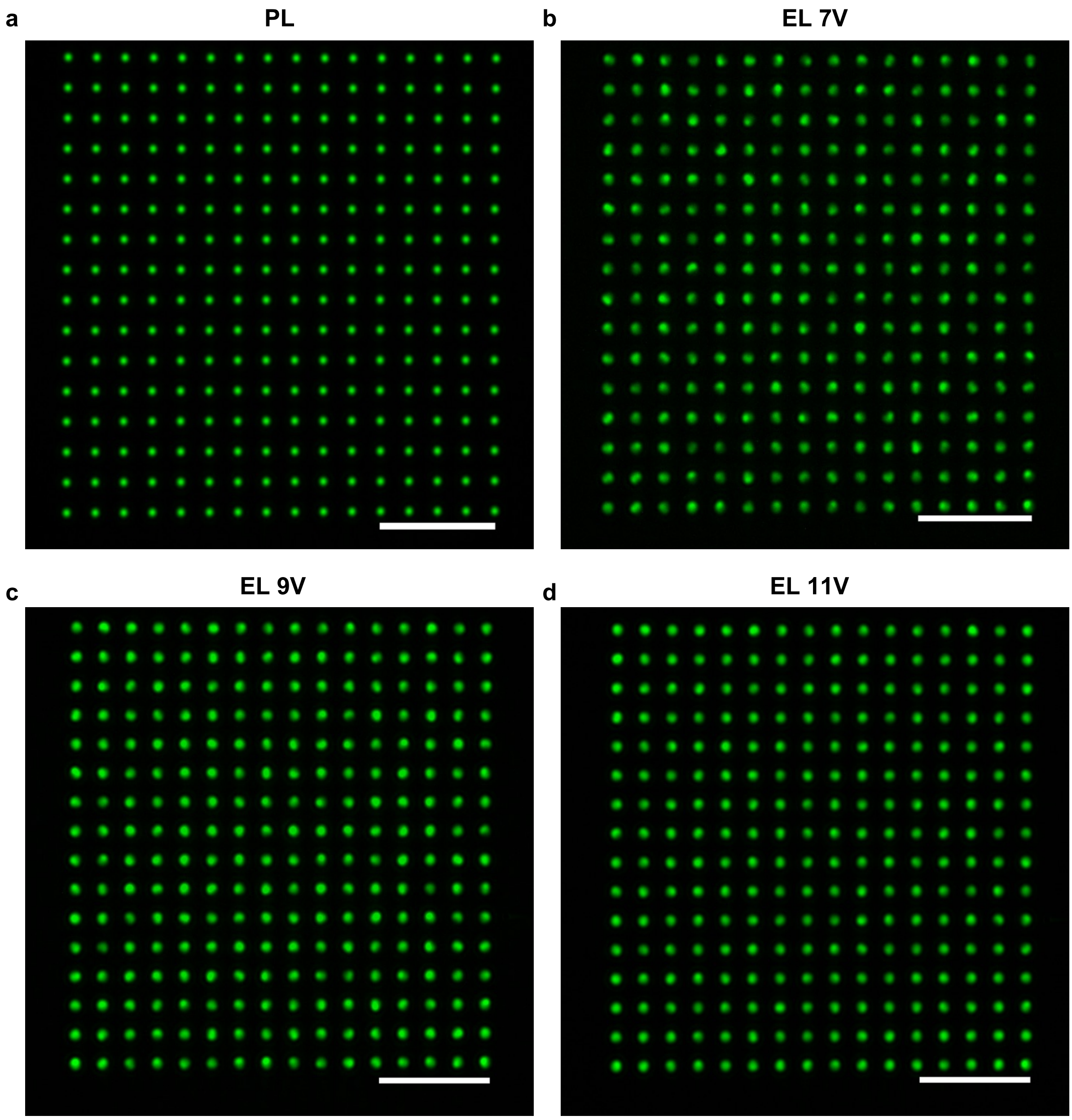

Supplementary Figure 19 | Emission non-uniformity in non-optimized devices. Emission non-uniformity in our initial nano-OLED devices, comparing the PL **(a)** and EL **(b-d)** micrographs at various voltages. The nano-OLED device contains square array of nanodisk pixels (diameter of 1 μm and periodicity of 5 μm). The asymmetric shape of individual pixels in the EL images suggest non-uniform charge injection due to imperfect HTL/EML stacking.

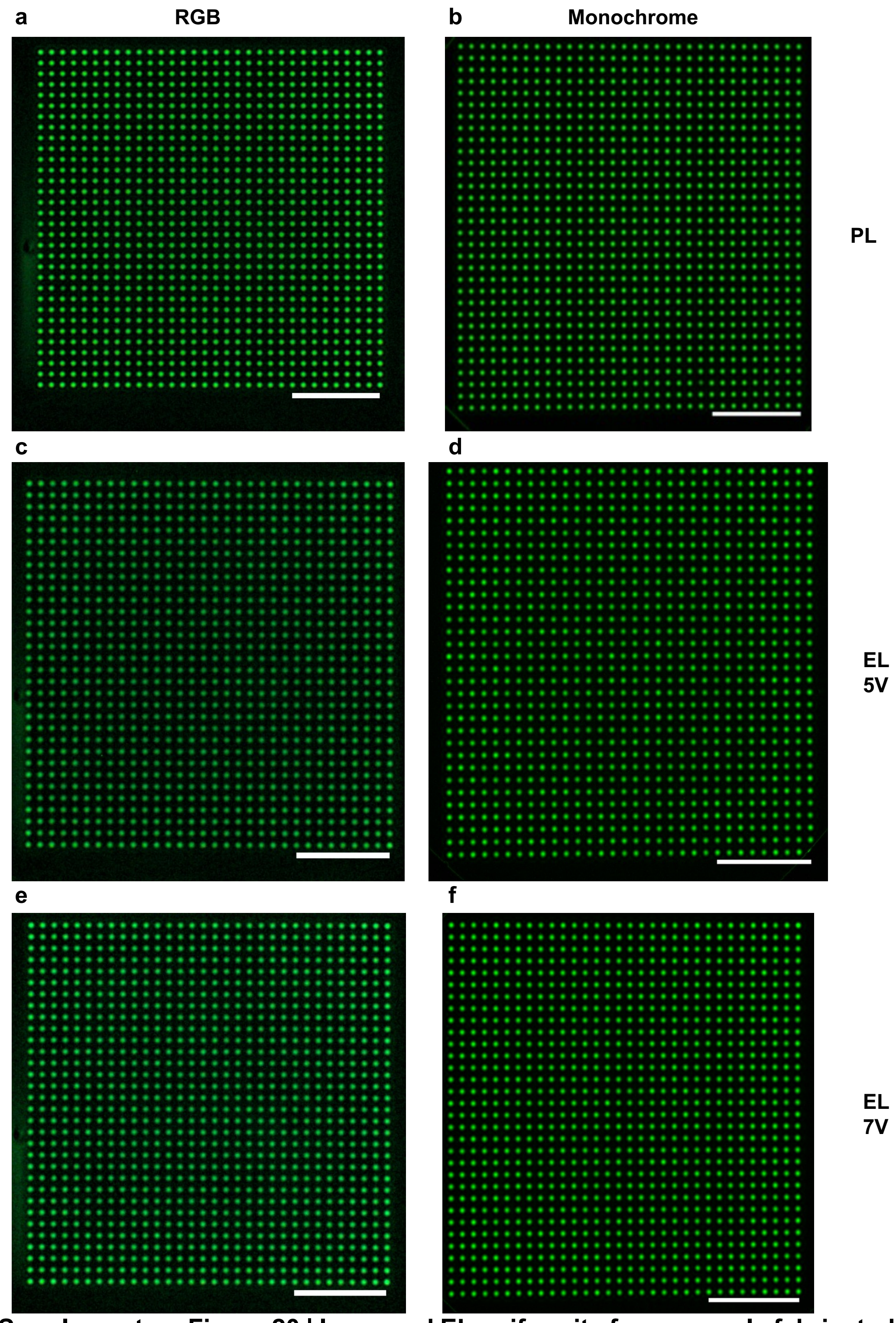

Supplementary Figure 20 | Improved EL uniformity for our newly fabricated nano-OLED devices (nanodisk array at 10,000 ppi, diameter of 500 nm and periodicity of 2.5 μm). Full color **(a,c,e)** and monochrome **(b, d, f)** micrographs taken from two independent sCMOS camera exhibit very uniform light intensity and chromaticity. All scale bars are 20 μm.

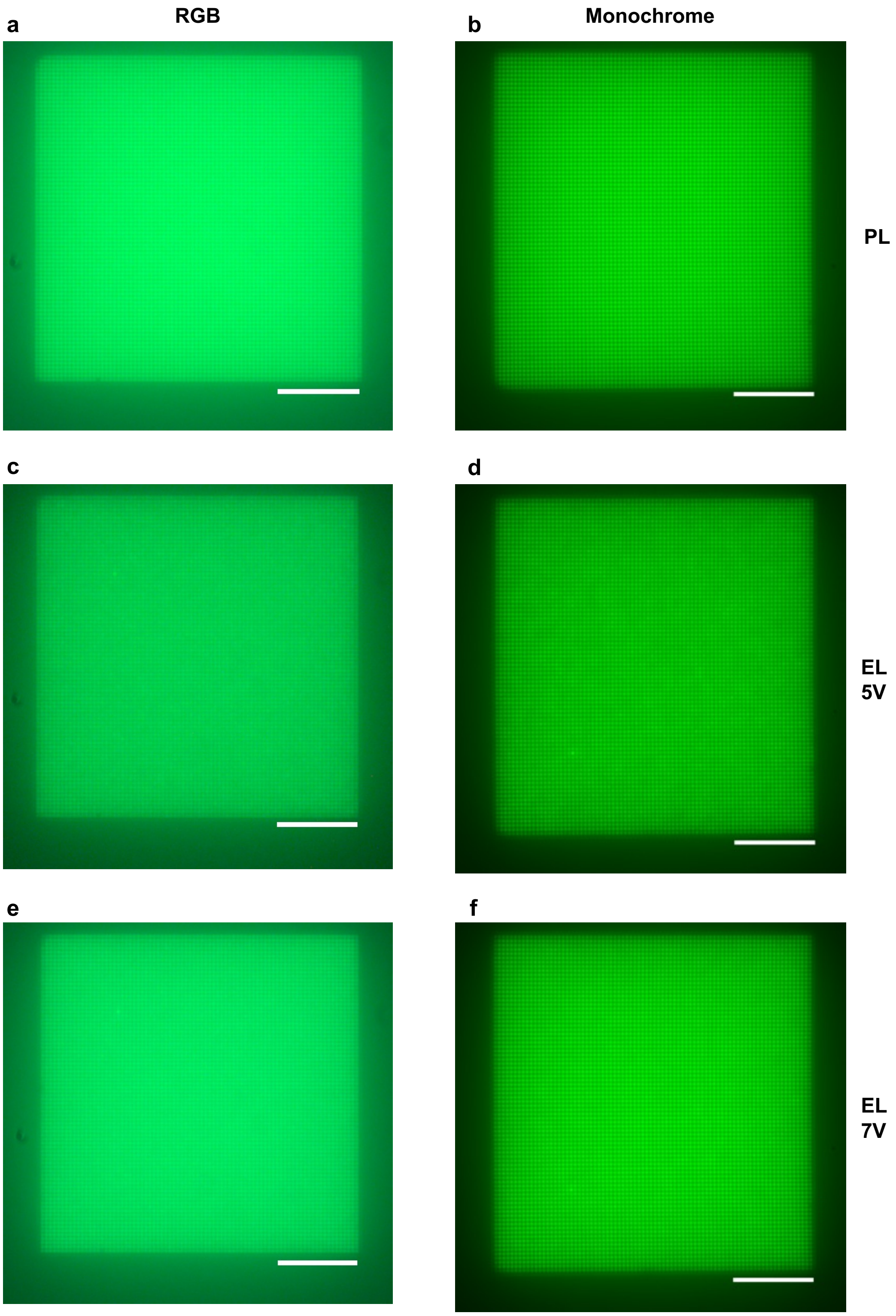

Supplementary Figure 21 | Improved EL uniformity for our newly fabricated nano-OLED devices (nanodisk array at 25,000 ppi, diameter of 200 nm and periodicity of 1 μm). Full color **(a,c,e)** and monochrome **(b, d, f)** micrographs taken from two independent sCMOS camera exhibit very uniform light intensity and chromaticity. All scale bars are 20 μm.

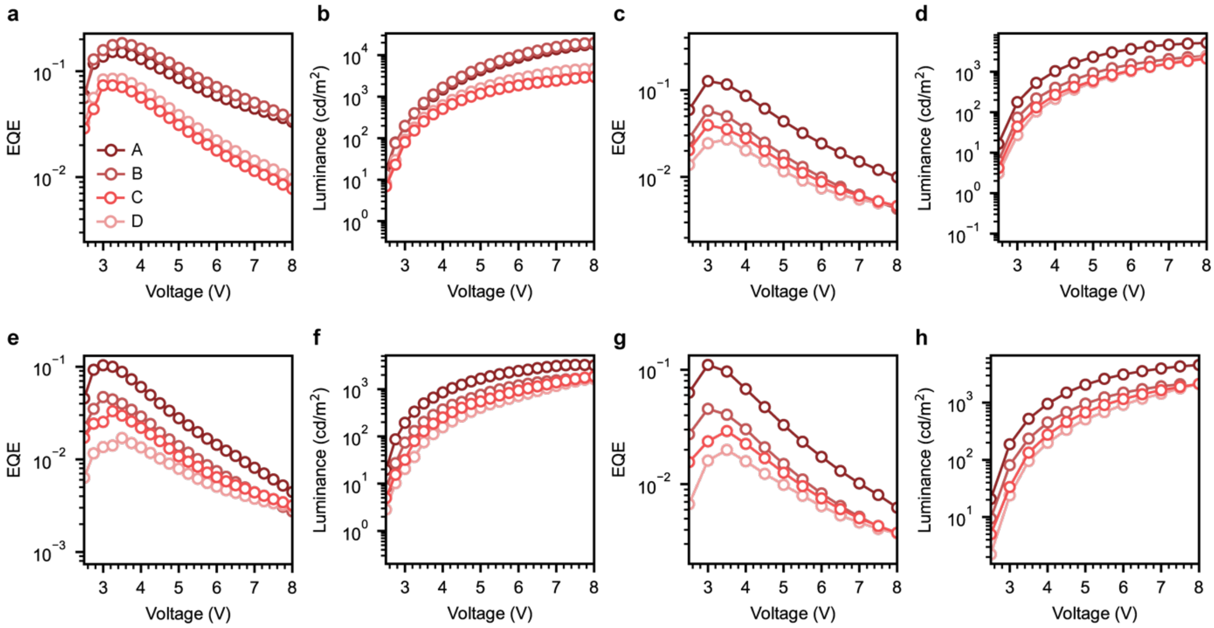

Supplementary Figure 22 | Size dependence of nano-OLED performance. EQE and luminance performance data for two batches of nano-OLED devices having constant void fraction of 60% (**a-d**) and 70% (**e-h**). For 60 % void fraction, the nanodisk diameters are as follows: A: 330 nm; B: 660 nm; C: 1320 nm; D: 2640 nm. For 70% devices, A: 288nm; B: 576 nm, C: 1159 nm, D: 2300nm.

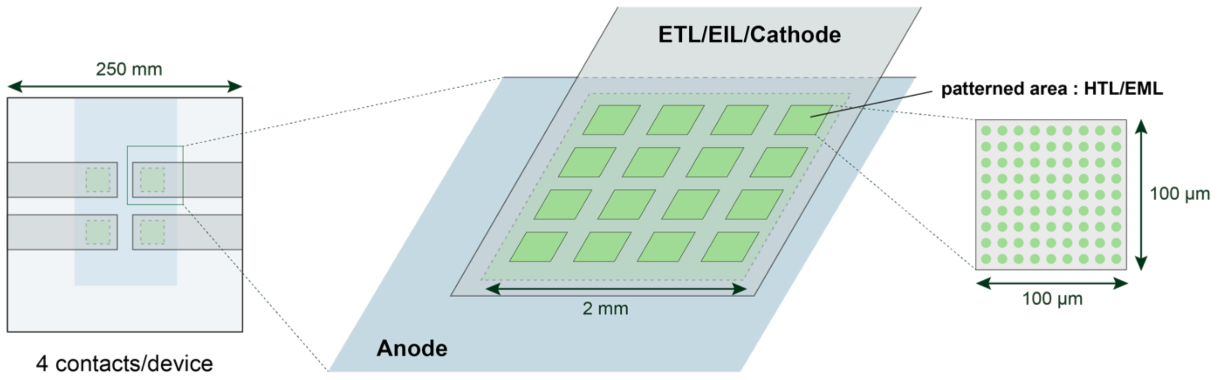

Supplementary Figure 23 | Schematic diagram illustrating the layout of a full nano-OLED device.

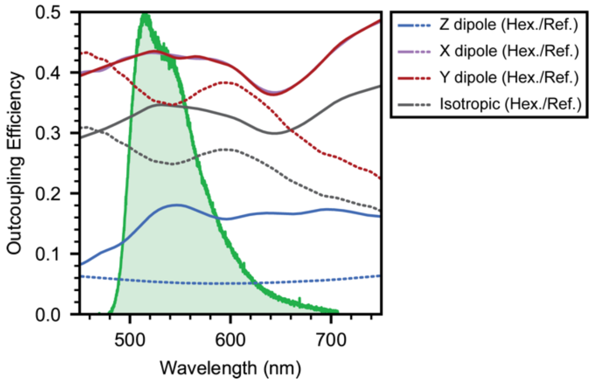

Supplementary Figure 24 | Simulations of outcoupling efficiency from nano-OLEDs Optical simulations for comparing the outcoupling efficiencies for the bulk thin-film (dashed lines) and nano-OLED (solid lines) devices, considering an x- (red), y- (purple), and z- (blue) oriented dipoles and the ensemble, isotropically oriented dipole (gray).

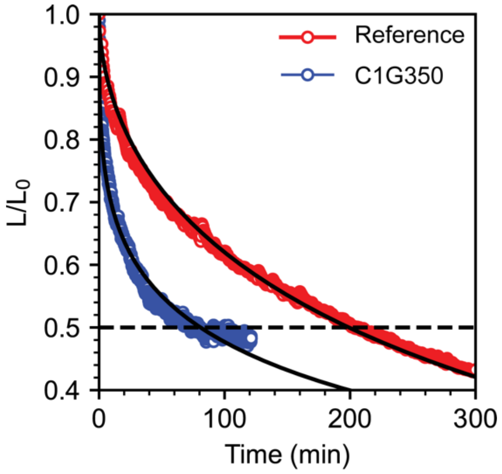

Supplementary Figure 25 | Nano-OLED operational lifetime. Normalized luminance decay (initial luminance $L_0 = 1{,}000$ cd m^{-2}) as a function of time stressed at constant current. The nano-OLED device considered here is comprised of square array of nanodisk OLED pixels, with diameter of 100 nm and periodicity of 350 nm. The black lines are the fittings with a stretched exponential decay model, showing LT50 values of 218 and 80 minutes for the bulk thin-film and nano-OLED devices, respectively.

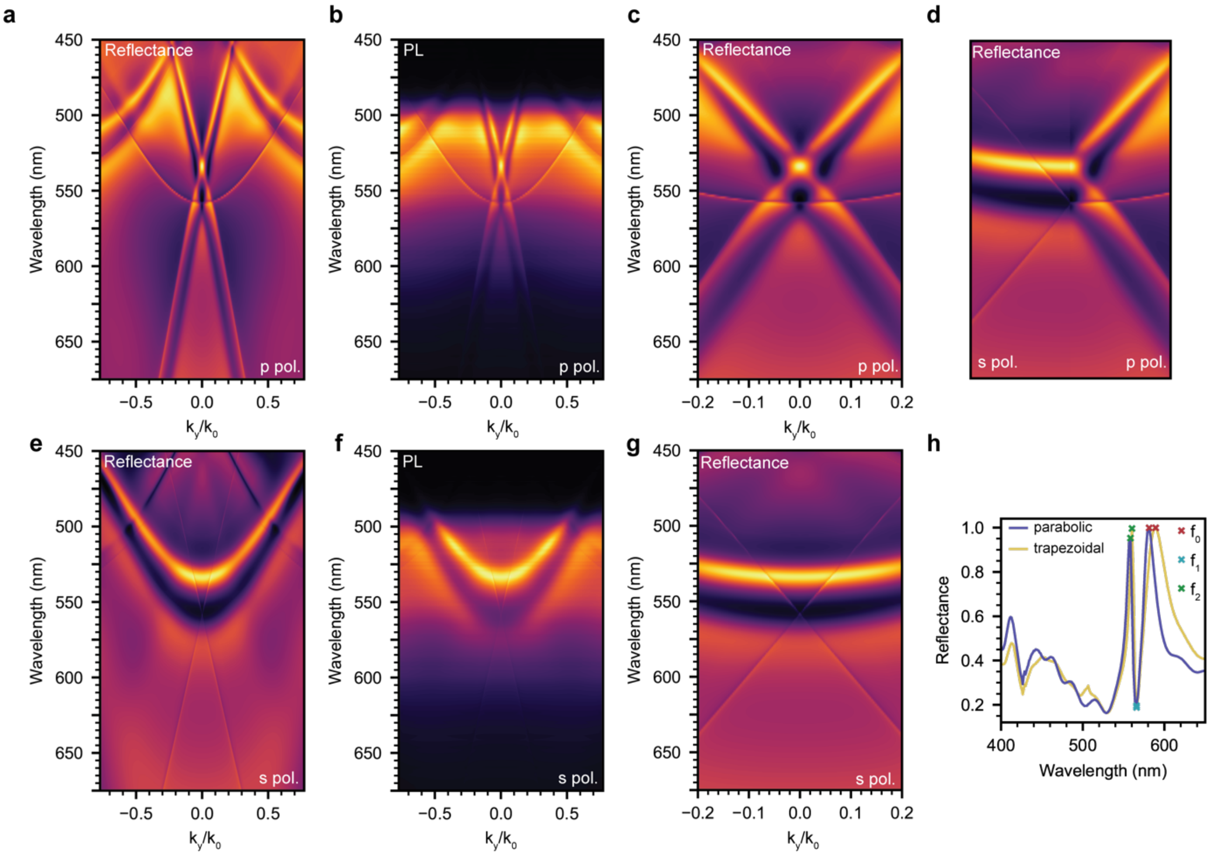

Supplementary Figure 26 | RCWA dispersion of nano-OLEDs. Simulated reflectance (**a, e)** and PL (**b, f**) dispersions for the nano-OLED pattern showed in Fig. 3a and Extended Fig. 4a. **c, d, g** magnified versions of **a** and **b** for the assignment of the resonant modes. **h** Reflectance responses at the Γ point for parabolic and trapezoidal height profiles. The three most relevant resonances are labelled as f_0, f_1 and f_2.

Force-field parameters for CBP

Supplementary Table 1 | CHARMM bonded parameters from the SwissParam server. The potential energy expression is $V_{bond} = k_b(b - b_0)^2$.

**Bond**	$k_b \left(\frac{\text{kcal}}{\text{mol} \cdot \text{Å}^2}\right)$	b_0(Å)
CB – CB	401.068	1.374
CB – C5A	438.634	1.372
C5A – C5B	512.256	1.377
C5B – CB	443.384	1.379
C5B – C5B	310.390	1.418
C5A – NPYL	453.459	1.364
NPYL – CB	406.609	1.388
CB – HCMM	381.853	1.084

Supplementary Table 2 | CHARMM angle parameters from the SwissParam server. The potential energy expression is $V_{angle} = k_\theta(\theta - \theta_0)^2$.

**Angle**	$k_\theta \left(\frac{\text{kcal}}{\text{mol} \cdot \text{rad}^2}\right)$	θ(deg)
CB – CB – CB	48.145	119.977
CB – CB – HCMM	40.517	120.571
CB – CB – C5A	34.400	111.243
C5A – CB – HCMM	50.520	121.238
CB – C5A – C5B	48.865	122.881
CB – C5A – NPYL	72.757	132.046
C5B – C5A – NPYL	58.508	107.255
C5A – C5B – CB	65.201	117.966
C5A – C5B – C5B	62.322	108.239
CB – C5B – C5B	61.459	136.087
C5B – CB – CB	30.441	112.567
C5B – CB – HCMM	37.638	121.446
C5A – NPYL – C5A	82.904	109.599
C5A – NPYL – CB	66.352	125.312
NPYL – CB – CB	77.579	114.622

Supplementary Table 3 | CHARMM dihedral parameters from the SwissParam server. The potential energy expression is $V_{dihedral} = k_\chi(1 + \cos(n\chi - \delta))$.

Dihedral	$k_\chi\left(\frac{\mathbf{kcal}}{\mathbf{mol}}\right)$	n	$\theta_0(\mathbf{deg})$
CB – CB – C5A – C5B	3.500	2	180.00
CB – CB – C5A – NPYL	3.500	2	180.00
CB – CB – CB – C5B	3.500	2	180.00
CB – CB – CB – HCMM	3.500	2	180.00
CB – CB – CB – CB	3.500	2	180.00
CB – C5A – C5B – CB	3.500	2	180.00
CB – C5A – C5B – C5B	3.500	2	180.00
CB – C5A – NPYL – C5A	2.000	2	180.00
CB – C5A – NPYL – CB	3.000	2	180.00
C5A – CB – CB – CB	3.500	2	180.00
C5A – CB – CB – HCMM	3.500	2	180.00
C5A – C5B – CB – CB	3.500	2	180.00
C5A – C5B – CB – HCMM	3.500	2	180.00
C5A – C5B – C5B – C5A	3.500	2	180.00
C5A – C5B – C5B – CB	3.500	2	180.00
C5A – NPYL – C5A – C5B	2.000	2	180.00
C5A – NPYL – CB – CB	3.000	2	180.00
C5B – C5A – CB – HCMM	3.500	2	180.00
C5B – C5A – NPYL – CB	3.000	2	180.00
C5B – CB – CB – HCMM	3.500	2	180.00
C5B – C5B – C5A – NPYL	3.500	2	180.00
C5B – C5B – CB – CB	3.500	2	180.00
C5B – C5B – CB – HCMM	3.500	2	180.00
CB – C5B – C5A – NPYL	3.500	2	180.00
CB – C5B – C5B – CB	3.500	2	180.00
NPYL – C5A – CB – HCMM	3.500	2	180.00
NPYL – CB – CB – CB	1.000	2	180.00
NPYL – CB – CB – HCMM	1.000	2	180.00
HCMM – CB – CB – HCMM	3.500	2	180.00

Supplementary Table 4 | CHARMM improper parameters from the SwissParam server. The potential energy expression is $V_{improper} = k_{\varphi}(\varphi - \varphi_0)^2$.

Improper	$\boldsymbol{k_{\varphi}}\left(\frac{\mathbf{kcal}}{\mathbf{mol \cdot rad^2}}\right)$	$\boldsymbol{\varphi_0}(\mathbf{deg})$
CB – CB – CB – HCMM	1.079	0.00
CB – C5A – CB – HCMM	0.576	0.00
C5A – NPYL – CB – C5B	0.720	0.00
C5B – C5B – C5A – CB	-0.792	0.00
C5B – CB – C5B – C5A	-0.792	0.00
C5A – NPYL – C5B – CB	0.720	0.00
CB – CB – C5A – HCMM	0.576	0.00
NPYL – C5A – C5A – CB	1.439	0.00
CB – CB – NPYL – CB	2.519	0.00
CB – CB – CB – CB	2.519	0.00
NPYL – C5A – CB – C5A	1.439	0.00
C5A – C5B – NPYL – CB	0.720	0.00
C5B – CB – C5A – C5B	-0.792	0.00
C5B – C5A – C5B – CB	-0.792	0.00
CB – CB – C5B – HCMM	0.864	0.00
CB – C5B – CB – HCMM	0.864	0.00

Supplementary Table 5 | CHARMM Lennard-Jones parameters from the SwissParam server. The potential energy expression is $V_{LJ} = \varepsilon_{ij}\left[\left(\frac{R_{\min_{ij}}}{r_{ij}}\right)^{12} - 2\left(\frac{R_{\min_{ij}}}{r_{ij}}\right)^{6}\right]$ where $\varepsilon_{ij} = \sqrt{\varepsilon_{ii}\varepsilon_{jj}}$ and $R_{\min_{ij}} = \frac{R_{\min_{ii}} + R_{\min_{jj}}}{2}$.

Atom Type	$\boldsymbol{\varepsilon}\left(\frac{\mathbf{kcal}}{\mathbf{mol}}\right)$	$\frac{\boldsymbol{R}_{\mathbf{min}}}{\mathbf{2}}(\text{Å})$
CB	0.070000	1.992400
C5A	0.068000	2.090000
C5B	0.068000	2.090000
NPYL	0.200000	1.850000
HCMM	0.022000	1.320000

Force-field parameters for $Ir(ppy)_3$

Supplementary Table 6 | Bonded parameters developed using ffTK. The potential energy expression is $V_{bond} = k_b(b - b_0)^2$.

Bond	$\boldsymbol{k_b}\left(\frac{\mathbf{kcal}}{\mathbf{mol \cdot Å^2}}\right)$	$\boldsymbol{b_0}(\text{Å})$
Iri – NIr	149.461	2.087
Iri – CIr	140.336	1.953
CIr – CC	298.287	1.476
CIr – CH	380.279	1.455
CNC – CH	281.128	1.400
CNC – NIr	118.460	1.360
CNC – CC	265.874	1.489
HC – CH	355.249	1.086
CH – CH	339.024	1.381
CH – CNH	431.333	1.378
CC – CH	299.500	1.311
HC – CNH	373.339	1.078
NIr – CNH	336.691	1.351

Supplementary Table 7 |. Angle parameters developed using ffTK. The potential energy expression is $V_{angle} = k_\theta(\theta - \theta_0)^2$.

Angle	$\boldsymbol{k_\theta}\left(\frac{\mathbf{kcal}}{\mathbf{mol \cdot rad^2}}\right)$	$\boldsymbol{\theta_0}(\mathbf{deg})$
CIr – Iri – NIr	43.398	98.547
NIr – Iri – NIr	31.300	119.526
CIr – Iri – CIr	14.643	119.685
Iri – CIr – CC	269.136	114.466
Iri – CIr – CH	293.221	133.112
CH – CIr – CC	251.860	119.195
NIr – CNC – CH	248.328	119.493
CC – CNC – CH	197.765	124.774
NIr – CNC – CC	266.370	115.156
CIr – CH – CH	239.826	127.461
CIr – CH – HC	87.068	118.730
CH – CH – HC	73.671	119.944
HC – CH – CNH	79.710	119.674

CNH – CH – CH	194.954	114.579
CIr – CC – CNC	254.494	116.347
CIr – CC – CH	237.674	124.785
CNC – CC – CH	227.789	122.612
Iri – NIr – CNC	220.288	116.054
Iri – NIr – CNH	270.119	128.340
CNC – NIr – CNH	194.960	119.611
CNC – CH – CH	148.655	120.276
HC – CH – CNC	78.926	119.547
CH – CH – CH	137.659	119.650
HC – CNH – NIr	87.999	115.076
HC – CNH – CH	91.153	119.503
NIr – CNH – CH	238.044	122.544
CC – CH – HC	77.091	121.043
CC – CH – CH	197.058	119.952

Supplementary Table 8 | Lennard-Jones parameters obtained from ffTK. The potential energy expression is $V_{LJ} = \varepsilon_{ij}\left[\left(\frac{R_{\min_{ij}}}{r_{ij}}\right)^{12} - 2\left(\frac{R_{\min_{ij}}}{r_{ij}}\right)^{6}\right]$ where $\varepsilon_{ij} = \sqrt{\varepsilon_{ii}\varepsilon_{jj}}$ and $R_{\min_{ij}} = \frac{R_{\min_{ii}} + R_{\min_{jj}}}{2}$.

**Atom Type**	$\boldsymbol{\varepsilon}\left(\frac{\mathbf{kcal}}{\mathbf{mol}}\right)$	$\frac{\boldsymbol{R_{\min}}}{\mathbf{2}}$ (Å)
CC	0.070000	1.992400
CH	0.070000	1.992400
CIr	0.070000	1.992400
CNC	0.070000	1.992400
CNH	0.070000	1.992400
HC	0.022000	1.320000
Iri	9.200000	1.392500
NIr	0.200000	1.850000

Pattern	Mean gap g, a (um)	a_{std} (um)
A11	5.923, 0.733	0.028
A22	5.961, 0.771	0.029
A33	6.013, 0.823	0.026
A44	6.069, 0.879	0.030
Ac1	6.073, 0.883	0.028

Supplementary Table 9 | Statistical analysis for gap distances at different areas. The values of mean gap distances and its standard deviation in μm for various different patterns across the entire substrate. As illustrated in Supplementary Fig. S10b. The mean values of substrate-stencil distance g and air gap distance a are extracted following $a = (g - s)$.

Sample	Max. EQE (%)	Max. Luminance (cd/m^2)
No etching (neat PEDOT:PSS)	18.6	40210
30 s O_2 RIE etching	20.1	56853
40 s O_2 RIE etching	18.3	43323
50 s O_2 RIE etching	20.5	14135

Supplementary Table 10 | Reference OLED performance and effects of PEDOT:PSS over-etching. Comparison of key device characteristics, maximum (Max.) EQE and luminance for the devices considered here.

REFERENCES

1. Zeng, S., Tian, T., Oh, J., Lin, Z.-H. & Shih, C.-J. Direct nanopatterning of complex 3D surfaces and self-aligned superlattices via molecular-beam holographic lithography. *Nat. Commun.* **16**, 3436 (2025).
2. Gottscho, R. A. Ion transport anisotropy in low pressure, high density plasmas. *J. Vac. Sci. Technol. B Microelectron. Nanometer Struct. Process. Meas. Phenom.* **11**, 1884–1889 (1993).
3. Rodina, A. V. & Efros, Al. L. Effect of dielectric confinement on optical properties of colloidal nanostructures. *J. Exp. Theor. Phys.* **122**, 554–566 (2016).
4. Wang, J., Gudiksen, M. S., Duan, X., Cui, Y. & Lieber, C. M. Highly Polarized Photoluminescence and Photodetection from Single Indium Phosphide Nanowires. *Science* **293**, 1455–1457 (2001).
5. Hanwell, M. D. *et al.* Avogadro: an advanced semantic chemical editor, visualization, and analysis platform. *J. Cheminformatics* **4**, 17 (2012).
6. Humphrey, W., Dalke, A. & Schulten, K. VMD: Visual molecular dynamics. *J. Mol. Graph.* **14**, 33–38 (1996).
7. Martínez, L., Andrade, R., Birgin, E. G. & Martínez, J. M. P ACKMOL : A package for building initial configurations for molecular dynamics simulations. *J. Comput. Chem.* **30**, 2157–2164 (2009).
8. Phillips, J. C. *et al.* Scalable molecular dynamics with NAMD. *J. Comput. Chem.* **26**, 1781–1802 (2005).
9. Brooks, B. R. *et al.* CHARMM : A program for macromolecular energy, minimization, and dynamics calculations. *J. Comput. Chem.* **4**, 187–217 (1983).
10. Bugnon, M. *et al.* SwissParam 2023: A Modern Web-Based Tool for Efficient Small Molecule Parametrization. *J. Chem. Inf. Model.* **63**, 6469–6475 (2023).

11. Mayne, C. G., Saam, J., Schulten, K., Tajkhorshid, E. & Gumbart, J. C. Rapid parameterization of small molecules using the force field toolkit. *J. Comput. Chem.* **34**, 2757–2770 (2013).

12. Essmann, U. *et al.* A smooth particle mesh Ewald method. *J. Chem. Phys.* **103**, 8577–8593 (1995).

13. Langevin P. On the theory of Brownian motion, C. R. Acad. Sci. (Paris) 1908, 146, 530.
